# Treatment of acute myeloid leukemia models by targeting a cell surface RNA-binding protein

**DOI:** 10.1038/s41587-025-02648-2

**Published:** 2025-04-23

**Authors:** Benson M. George, Maria Eleftheriou, Eliza Yankova, Jonathan Perr, Peiyuan Chai, Gianluca Nestola, Karim Almahayni, Siân Evans, Aristi Damaskou, Helena Hemberger, Charlotta G. Lebedenko, Justyna Rak, Qi Yu, Ece Bapcum, James Russell, Jaana Bagri, Regan F. Volk, Malte Spiekermann, Richard M. Stone, George Giotopoulos, Brian J. P. Huntly, Joanna Baxter, Fernando Camargo, Jie Liu, Balyn W. Zaro, George S. Vassiliou, Leonhard Möckl, Jorge de la Rosa, Ryan A. Flynn, Konstantinos Tzelepis

**Affiliations:** 1 Stem Cell Program and Division of Hematology/Oncology, Boston Children’s Hospital, Boston, MA USA; 2 Department of Medical Oncology, Dana-Farber Cancer Institute, Boston, MA USA; 3 Cambridge Stem Cell Institute, University of Cambridge, Cambridge, UK; 4 Department of Haematology, University of Cambridge, Cambridge, UK; 5 Milner Therapeutics Institute, University of Cambridge, Cambridge, UK; 6 Department of Physics, Friedrich-Alexander University of Erlangen–Nuremberg, Erlangen, Germany; 7 Max Planck Institute for the Science of Light, Erlangen, Germany; 8 Department of Pharmaceutical Chemistry, Cardiovascular Research Institute, University of California, San Francisco, San Francisco, CA USA; 9 Institute for Stem Cell Biology and Regenerative Medicine, Stanford, CA USA; 10 Faculty of Medicine 1, Friedrich-Alexander-Universität Erlangen–Nürnberg, Erlangen, Germany; 11 Faculty of Sciences, Department of Physics, Friedrich-Alexander-Universität Erlangen–Nürnberg, Erlangen, Germany; 12 Cambridge Institute for Therapeutic Immunology and Infectious Disease, University of Cambridge, Cambridge, UK; 13 Department of Stem Cell and Regenerative Biology, Harvard University, Cambridge, MA USA; 14 Harvard Stem Cell Institute, Harvard University, Cambridge, MA USA; 15 Wellcome Trust Sanger Institute, Hinxton, UK; 16 Present Address: Cambridge Institute of Science, Altos Labs, Cambridge, UK

**Keywords:** Cancer, Proteomics

## Abstract

Immunotherapies for acute myeloid leukemia (AML) and other cancers are limited by a lack of tumor-specific targets. Here we discover that RNA-binding proteins and glycosylated RNAs (glycoRNAs) form precisely organized nanodomains on cancer cell surfaces. We characterize nucleophosmin (NPM1) as an abundant cell surface protein (csNPM1) on a variety of tumor types. With a focus on AML, we observe csNPM1 on blasts and leukemic stem cells but not on normal hematopoietic stem cells. We develop a monoclonal antibody to target csNPM1, which exhibits robust anti-tumor activity in multiple syngeneic and xenograft models of AML, including patient-derived xenografts, without observable toxicity. We find that csNPM1 is expressed in a mutation-agnostic manner on primary AML cells and may therefore offer a general strategy for detecting and treating AML. Surface profiling and in vivo work also demonstrate csNPM1 as a target on solid tumors. Our data suggest that csNPM1 and its neighboring glycoRNA–cell surface RNA-binding protein (csRBP) clusters may serve as an alternative antigen class for therapeutic targeting or cell identification.

## Abstract (web summary)

An RNA-binding protein on leukemia cells provides an effective target in mouse models.

## Main

In recent years, there has been a surge in new therapies that target cell surface molecules, including monoclonal antibodies, antibody–drug conjugates, bispecific antibodies and chimeric antigen receptor T cells^1–4^. These approaches have improved overall survival in a variety of cancers, including non-Hodgkin lymphoma, breast cancer and acute leukemia. While the molecular toolkit for targeting cell surface molecules has rapidly expanded, there remain few clinically actionable targets to leverage these approaches. There have been efforts to better understand and characterize cell surface proteins that are differentially expressed between healthy and cancer states using techniques like proteomics, transcriptomics and phage display^5–7^. Despite the emergence of these unbiased techniques, AML has remained challenging to target with surface-directed therapies, as many highly expressed antigens on AML cells are also present on critical healthy tissue, including hematopoietic stem cells.

Anti-CD33 molecules, like the antibody–drug conjugate gemtuzumab ozogamicin^8^, have seen some success; however, high expression of CD33 on healthy myeloid cells and hepatic sinusoidal endothelial cells has been associated with severe hematological and hepatic toxicity, respectively^9^. Newer approaches studying both the expression and the physical conformation of proteins on the cell surface have demonstrated promising results, such as an antibody fragment targeting an AML-specific conformation of integrin β_2_ (ref. ^10^). Another approach that has gained traction has been targeting HLA-restricted peptides, such as WT1 (ref. ^11^); however, this approach is limited to a fraction of patients who express specific HLA subtypes. Now there are methods to genetically delete surface proteins from healthy hematopoietic tissue to allow AML cytotoxicity with limited off-target effects^12^; however, this may be technically cumbersome and costly. Although the majority of clinical efforts have focused on canonical cell surface proteins^13^, such as those containing a transmembrane domain, there appears to be a need to expand our scope.

There are now three examples of RBPs as surface proteins in preclinical cancer models: PABP1 (ref. ^14^), nucleolin^15–18^ and U5-snRNP200 (ref. ^19^). There are multiple examples of mutated and dysregulated RBPs in AML, such as SF3B1, U2AF1, SRSF2 and NPM1. In adult AML, nearly 30% of all disease occurrences and approximately 60% of those with a normal karyotype are driven by mutations in *NPM1* (refs. ^20,21^). In these patients, C-terminal mutations of NPM1 cause the replacement of a nucleolar localization signal with a novel nuclear export sequence, leading to cytoplasmic localization of the mutant protein (NPM1c)^22^. However, it has been unclear whether NPM1 (either wild type (WT) or mutated) is present on the cell surface. Outside of leukemia, autoantibodies in the sera from patients with prostate cancer were shown to react with NPM1, while those from healthy controls did not^23^, indicating potential aberrant cell surface localization leading to immune recognition.

Here, we provide evidence that NPM1 is expressed on the cell surface in a tumor-selective manner. csNPM1 is present on a diverse set of clinically relevant human and mouse leukemia models and forms nanoscale clusters in physical proximity to other csRBPs and glycoRNAs^24,25^. We demonstrate that csNPM1 is expressed at increased levels in primary AML in comparison to healthy hematopoietic stem and progenitor cells (HSPCs). We also optimize a new mouse immunoglobulin (Ig)G2a antibody (mAb2) targeting NPM1, which does not cause toxicity in healthy mice in vivo. mAb2 shows preferential targeting of murine leukemia stem cells (LSCs) and increases survival in murine models of AML while restoring healthy hematopoiesis. csNPM1 is also present on solid cancers cell lines, and, using in vivo models, we find that targeting csNPM1 has anti-tumor activity in murine models of prostate and colorectal (csNPM1-high) cancer but not melanoma (csNPM1 absent). Collectively, we provide evidence of an RBP that can localize selectively to the surface of cancer cells, which could have important therapeutic implications.

## Results

### Full-length NPM1 is presented on the surface of living cells

**Fig. 1:**
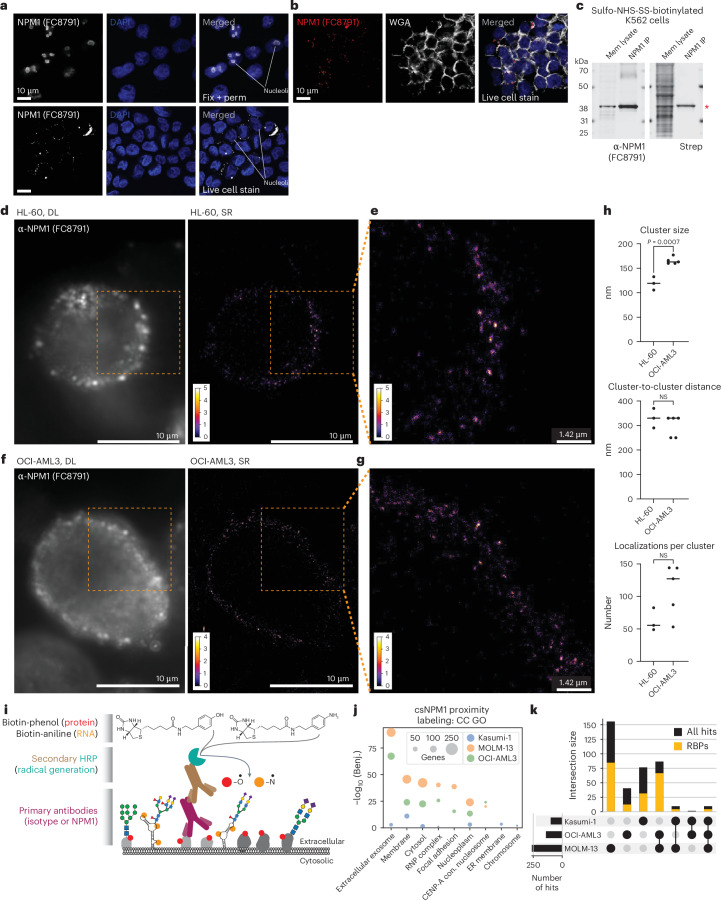
Full-length NPM1 forms nanoclusters on the surface of cells with other RBPs. **a**, Confocal microscopy images of OCI-AML3 cells presented as one *z* slice (all images are *z* slices unless otherwise specified). Cells in the top row were stained with the anti-NPM1 (FC8791) antibody after fixation and permeabilization (fix + perm), and cells in the bottom row were stained while alive. DAPI (4′,6-diamidino-2-phenylindole) is shown in blue. Scale bar, 10 μm. Nucleoli are indicated with white lines. Representative of three individual replicates. **b**, Confocal microscopy images of OCI-AML3 cells stained alive with anti-NPM1 (FC8791) antibody and biotinylated WGA. DAPI shown in blue. Representative of three individual replicates. **c**, Western blot of K562 cells first labeled with sulfosuccinimidyl-20(biotinamido)ethyl-1,3-dithiopropionate (sulfo-NHS-SS-biotin). NPM1 immunoprecipitation (IP; left) or streptavidin (strep) immunoprecipitation (right) was performed, and blots were stained with anti-NPM1 antibody or streptavidin. Molecular weights are shown in kDa. Red asterisks mark the expected weight of full-length NPM1. Representative of three individual replicates. Mem, membrane. **d**, HL-60 cells stained live with anti-NPM1 antibody and subsequently fixed. Shown are diffraction-limited (DL) widefield microscopy images (left) or SR reconstructions (right), presented as two-dimensional (2D) histograms (bin size, 17.7 nm). For SR images, the calibration bar indicates the number of localizations per histogram bin. **e**, A magnified view of the indicated region in **d**. **f**, OCI-AML3 cells stained and analyzed as in **d**. **g**, A magnified view of the indicated region in **f**. **h**, Quantification of cluster size in nanometers, cluster-to-cluster distance (nm) and localizations per cell, for each cell. Median value is shown as a horizontal bar. Unpaired, two-sided *t*-tests were performed to evaluate the significance; NS, not significant. **i**, Schematic of HRP-based cell surface proximity labeling. **j**, GO CC analysis of csNPM1 proximity labeling MS data. The top four terms across the three cell lines were intersected, and the union is shown; significance (*y* axis) and set size (circle size) are plotted. ER, endoplasmic reticulum; Benj., Benjamini; CENP-A, centromere protein A; con., containing. **k**, Intersection analysis of the proximity labeling MS data. For each bar, the number of RBPs is overlaid in orange. Source data

**Extended Data Fig. 1:**
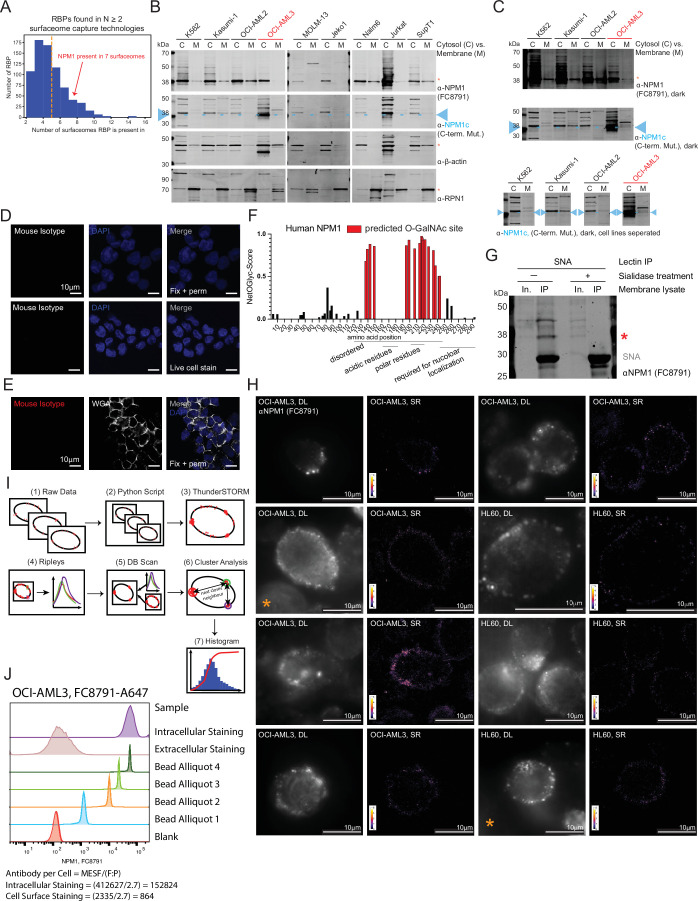
Organization of csNPM1 by confocal and super-resolution microscopy. **a**. Histogram plotting the number of each RBP (y-axis) that was found in a given set of cell surface proteomics experiments (surfaceomes, x-axis) as curated in^26^. **b**. Western blot analysis of cytosolic (C) or membrane (M) lysates from 9 human leukemia cell lines. Red asterisks denote the molecular weight of the target protein. The subtle difference in NPM1c’s molecular weight is highlighted by a blue dashed line and arrows. **c**. Higher exposure western blot panels from (B). Breakout panels below highlight the specific migration of NPM1c at the blue arrow. This band is only present in the OCI-AML3 cell line. **d**. Confocal microscopy images of OCI-AML3 cells as in Fig. 1a, here stained with the mouse isotype antibody. Scale bars are 10 μm. **e**. Confocal microscopy images of OCI-AML3 cells as in Fig. 1b, here stained with the mouse isotype antibody and biotinylated WGA. Scale bars are 10 μm. **f**. Histogram of each amino acid of human NPM1 scored by the NetOGlyc4.0 algorithm. Residues predicted to be a site of O-GalNAc modification are highlighted in red. Protein domains of NPM1 are annotated as defined by the Uniprot ID P06748. **g**. Western blot analysis of Lectin IP (SNA) from membrane lysates of K562 cells treated with or without sialidase. Input and IP samples were blotted for NPM1. Expected molecular weights of NPM1 is indicated with a red asterisk. **h**. Individual DL (left columns, grayscale) and SR (right columns, colored scales) images of each individual cell. Scale bars are noted on each image. For SR panels, individual color bars are provided to indicate the relative signal intensity. Two cells have an orange asterisk in the bottom left corners, indicating these are the cells presented in Fig. 1. **i**. Schematic of the experimental, data acquisition, and data analysis pipeline used to collect and analyze SR reconstructions. **j**. Quantitative flow cytometry of OCI-AML3 cells to quantify the amount of intracellular and cell surface NPM1. MESF is a measure of fluorescence intensity on the flow cytometer. F:P is the ratio of AF647 dyes per antibody molecule of the FC8791 stock that was used. Source data

Our examination of cell surface proteomes for csRBPs^26^ identified NPM1 as a putative cell surface protein, with at least seven datasets showing NPM1 enrichment on the cell surface (Extended Data Fig. 1a). Because NPM1 mislocalization is a driver of leukemia, we first examined the subcellular profile of NPM1 across a panel of nine human myeloid and lymphoid leukemia cell lines (Extended Data Fig. 1b,c), one of which harbors the NPM1c mutation (OCI-AML3). We isolated soluble cytosol and crude membrane fractions and assayed for cytosolic β-actin and endoplasmic reticulum membrane-tethered ribophorin I (RPN1), confirming efficient separation of these pools (Extended Data Fig. 1b,c). Blotting with an anti-NPM1 (FC8791) antibody produced a robust band corresponding to NPM1 from the cytosolic and membrane-enriched fraction of most cell lines tested (Extended Data Fig. 1b,c). Because OCI-AML3 harbors the C-terminal mutated form of NPM1 (NPM1c), we examined its cellular distribution using a commercially available anti-NPM1c antibody. We found that the NPM1c signal was detected in both the cytosol and the membrane fraction of OCI-AML3 cells (Extended Data Fig. 1b,c). We next tested whether the WT antibody could bind to the surface of live cells. To directly visualize the cell surface localization of NPM1, we performed confocal imaging of OCI-AML3 and found that, when applied to live cells, anti-NPM1 (FC8791) binds the cell surface in distinct puncta (Fig. 1a and Extended Data Fig. 1d). To demonstrate the specificity of the antibody, we performed intracellular immunofluorescence on fixed cells, resulting in the expected nucleolar predominance of NPM1 (Fig. 1a and Extended Data Fig. 1d). To confirm the cell surface pattern seen when staining live cells, we repeated the experiment with wheat germ agglutinin (WGA), which is a lectin that binds cell surface glycans. We observed robust WGA cell surface staining with colocalized anti-NPM1 (FC8791) signal (Fig. 1b and Extended Data Fig. 1e), further confirming NPM1 cell surface presentation.

To assess whether full-length NPM1 or a peptide fragment was presented on the cell surface, we used a sequential biochemical enrichment strategy. Live cell labeling with sulfo-NHS-SS-biotin, followed by membrane isolation, anti-NPM1 (FC8791) immunoprecipitation and finally western blotting resulted in robust enrichment of NPM1 from K562 membrane fractions (Fig. 1c, left). Using a streptavidin detection reagent on the same material resulted in a specific band migrating at the same molecular weight as NPM1 (Fig. 1c, right), supporting the presence of full-length csNPM1 rather than major histocompatibility complex presentation, as shown in other cases^27^. As many cell surface proteins are glycosylated, we next investigated whether NPM1 was similarly modified. While NPM1 has no *N*-glycan sequons, it does have many predicted sites for *O*-linked *N*-acetylgalactosamine (Extended Data Fig. 1f). Furthermore, we selectively enriched NPM1 from membrane lysates with a sialic acid-binding lectin (Extended Data Fig. 1g), suggesting that NPM1 can become glycosylated, consistent with a previous report^28^.

### Super-resolution localization of csNPM1

The presence of full-length csNPM1 and the punctate pattern observed with confocal microscopy in Fig. 1a motivated a more detailed investigation of its cell surface organization. We performed super-resolution (SR) single-molecule localization microscopy (Extended Data Fig. 1h) using a primary conjugated anti-NPM1 (FC8791) antibody for staining HL-60 (NPM1-WT) and OCI-AML3 (NPM1c) cells (Fig. 1d–h and Extended Data Fig. 1h). Staining live cells as in Fig. 1a and imaging with diffraction-limited widefield epifluorescent microscopy yielded an expected punctate pattern of csNPM1 (Fig. 1d,f). We obtained SR reconstructions for a total of five OCI-AML3 cells and three HL-60 cells (Supplementary Table 1 and Extended Data Fig. 1h,i). In both AML models, the SR reconstructions showed csNPM1 forming distinct clusters of apparently uniform size and regular patterning (Fig. 1d–g). We implemented a semi-automated cluster analysis workflow (Methods and Extended Data Fig. 1i). The diameter of csNPM1 clusters was on average 119 nm and 165 nm in HL-60 and OCI-AML3 cells, respectively (Fig. 1h). We then determined the number of localizations observed per cluster and the cluster-to-cluster distance. Across both cell lines, clusters were regularly spaced at approximately 300–330 nm apart (Fig. 1h), while the number of localizations was less uniform with clusters of two types: one with 50–80 and a second with 120–140 localizations per cluster (Fig. 1h). Using the SR data, we calculated the approximate number of antibodies bound per cell, finding ~1,000–2,400 and ~3,000–24,000 per cell on HL-60 and OCI-AML3 cells, respectively (Supplementary Table 1). A similar number was obtained by an orthogonal method using quantitative flow cytometry (Extended Data Fig. 1j). Together, the overall distribution of csNPM1 is reminiscent of a tessellated pattern on the cell surface.

### Molecular neighborhoods of csNPM1

**Extended Data Fig. 2:**
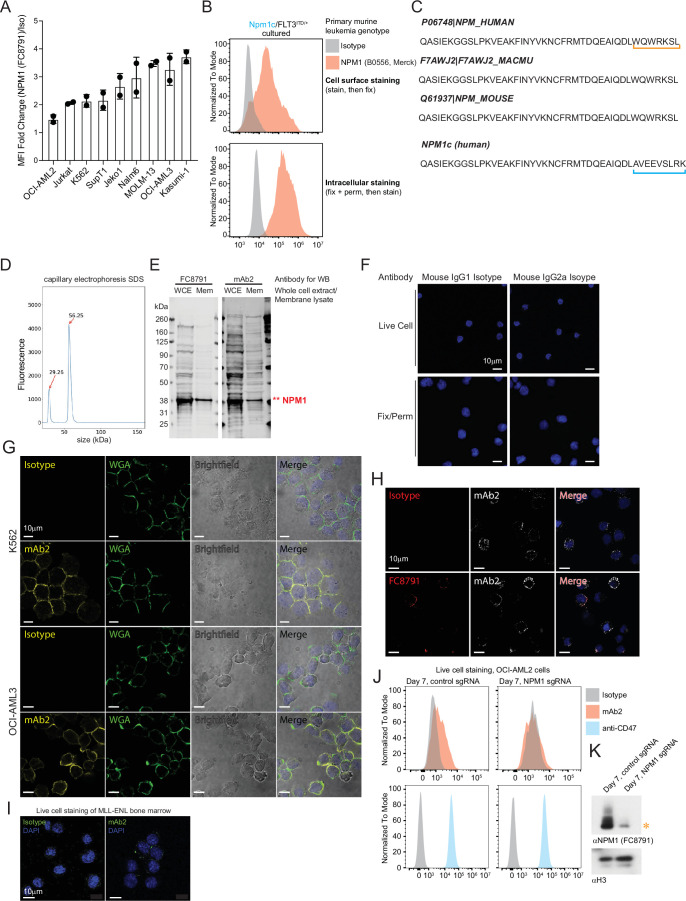
Characterization of novel anti-NPM1 antibody mAb2. **a**. Mean fluorescence intensity (MFI) analysis of 9x human leukemia cell lines.) **b**. Histograms of flow cytometry of the *Npm1c/Flt3*^*ITD/+*^ model isolated from bone marrow lysates and then grown *ex vivo* for 7 days. The top row is flow cytometry on live cells, the bottom cellswere permeabilized and fixed before adding the anti-NPM1 or isotype antibodies. **c**. Amino acid sequence comparison of the C-terminal domain of NPM1-WT (43 amino acids shown) from *Homo sapiens* (human), *Rhesus Macaque* (macmu), and *Mus musculus* (mouse), as well as the NPM1c mutant. The NPM1c 45 amino acids were used to identify the scFv mAb2 is designed on. **d**. Histogram of a denaturing (SDS) capillary electrophoresis run of the expressed mAb2. **e**. Western blot analysis of whole cell extract (WCE) or crude membrane fractions (Mem) from K562 cells. Membranes were stained with either FC8791 or mAb2. **f**. Confocal microscopy images of OCI-AML3 cells. Top row is live cell stained while the bottom row is stained on fixed and permeabilized cells with antibodies indicated. DAPI shown in blue. Scale bars are 10 μm. **g**. Confocal microscopy images of OCI-AML3 and K562 cells. Cells were stained either with an isotype control or mAb2 (yellow) and a co-stain with biotinylated WGA (green). Brightfield images are also shown with a triple merge (last column). DAPI shown in blue. Scale bars are 10 μm. **h**. Confocal microscopy images of OCI-AML3 cells. Cells were stained either with an isotype control or anti-NPM1 (FC8791, red) and a co-stain with mAb2 (red). The third column is a merged presentation to highlight overlapping signals. DAPI shown in blue. Scale bars are 10 μm. **i**. Confocal microscopy images of primary *MLL-ENL/Flt3*^*ITD/+*^ bone marrow cells. Cells were stained either with an isotype control or mAb2 (green). DAPI shown in blue. Scale bars are 10 μm. **j**. Flow cytometry analysis of live OCI-AML2 cells that have been subjected to genetic ablation of *NPM1* and stained with isotype (control, gray), mAb2 (red), or anti-CD47 (blue). **k**. Western blot analysis of native and *NPM1* knockout cells blotting for NPM1 or histone H3 (H3) as a loading control. **l**. Confocal microscopy images of primary MLL-ENL/Flt3^*ITD/+*^ bone marrow cells. Cells were stained either with an isotype control or mAb2 (green). DAPI shown in blue. Scale bars are 10 μm. Source data

Aside from rare examples, RBPs like NPM1 are not commonly thought to occupy the surface of living cells. Our recent work has begun to explore this concept more generally^26^; however, there remains an overall lack of molecular understanding of what factors may associate with cell surface RBPs. To address this, we adapted a strategy previously used to label glycoRNAs^24^, csRBPs^26^ and other proteins^29^ in proximity to glycan ligands. Using a primary antibody specific to csNPM1, a secondary antibody conjugated to horseradish peroxidase (HRP) was applied, which activates biotin-phenol (protein labeling) or biotin-aniline (RNA labeling) to label cell surface molecules in the presence of hydrogen peroxide (Fig. 1i and Supplementary Table 2). To ensure robust recovery of proximal proteins, we selected the three AML models with the highest csNPM1 mean fluorescence intensity (MFI) fold change over isotype: MOLM-13, OCI-AML3 and Kasumi-1 cells (Extended Data Fig. 2a). After capture of the biotinylated proteins and analysis of those hits enriched over the isotype labeling, we examined the enriched Gene Ontology (GO) cellular compartment (CC) terms. Membrane terms were highly enriched, suggesting that our approach was overall successful for assaying components of the cell surface (Fig. 1j). In addition, other GO terms such as cytosol, ribonucleoprotein (RNP) complex and nucleoplasm were also enriched (Fig. 1j). At the individual gene level, we found that MOLM-13, OCI-AML3 and Kasumi-1 cells had many unique hits (Fig. 1k, left three bars), and RBPs comprised 61.1%, 60.5% and 71% of the proteins proximal to csNPM1 (Fig. 1k). Notably, NPM1 was found in all three proximity labeling datasets as anticipated from our data above (Supplementary Table 2). Hits found in common between the AML models tested were more likely to be RBPs, as 75.9% of hits found proximal to csNPM1 in at least two cell lines were annotated RBPs (Supplementary Table 2). Together, these data suggest that csNPM1 clusters into distinct RBP-enriched nanodomains on the surface of leukemia cells.

### csNPM1 is expressed in human and murine models of AML

**Fig. 2:**
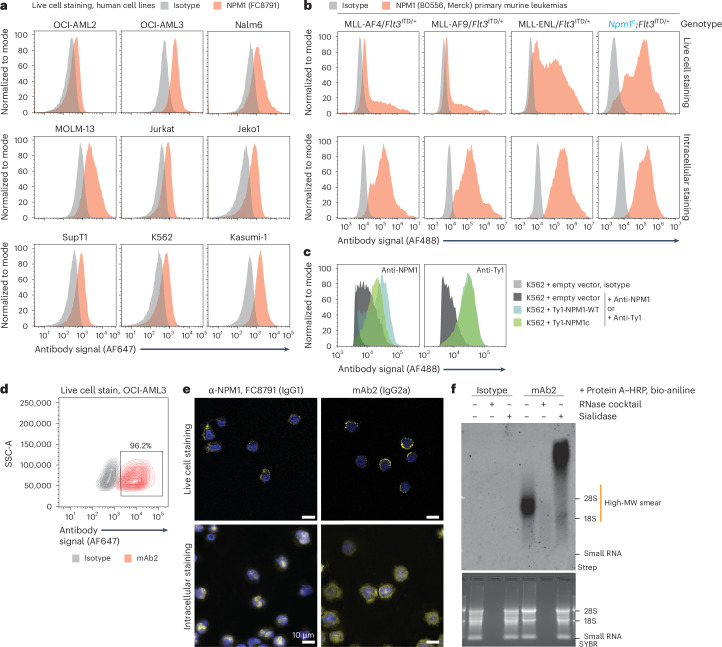
Commercial and new anti-NPM1 antibodies detect csNPM1 across many human and murine AML models. **a**, Histograms of live cell flow cytometry of nine human leukemia cell lines stained with an isotype (gray) or the commercially available anti-NPM1 FC8791 (orange) antibody. AF, Alexa Fluor. **b**, Histograms of flow cytometry of four primary murine leukemias isolated from BM-derived cells. AML-driving mutations are noted; all models are on a *Flt3*^ITD/+^ background. The top row shows flow cytometry on the surface, and the bottom shows intracellular staining of cells that were first fixed and permeabilized before adding the anti-NPM1 (B0556) or isotype antibodies. **c**, Histograms of live cell flow cytometry on K562 cells that were transduced with an over expression plasmid containing no complementary DNA (cDNA) (empty vector) or cDNAs encoding Ty1-tagged NPM1-WT (Ty1-NPM1-WT) or the NPM1c mutant (Ty1-NPM1c). Cells were stained either with anti-NPM1 (B0556, left) or anti-Ty1 (right) antibodies. **d**, Contour plot of OCI-AML3 cell live staining with the mAb2 (red) or isotype (gray) antibody. Cells shifted above the isotype are boxed, and the total percent of this population is noted. **e**, Confocal microscopy images of OCI-AML3 cells. Cells in the top row were stained with antibodies while they were alive, and those in the bottom row were first fixed with formaldehyde, permeabilized and then stained with antibodies: anti-NPM1 FC8791 (left) and mAb2 (right). All cells were counterstained with DAPI in blue. Scale bars, 10 μm. Representative of three individual replicates. **f**, Cell surface proximity labeling assisted by protein A–HRP and biotin-aniline (bio-aniline) to label cell surface RNAs. Isotype or mAb2 antibodies were used as anchors, and the resulting total RNA (SYBR Gold signal, SYBR) was analyzed on a northern blot, detecting biotinylated species (streptavidin IR800, strep). A high-molecular-weight (MW) smear is highlighted (orange) that is sensitive to RNase and sialidase treatment. Representative of three individual replicates. Source data

We next assessed the levels of csNPM1 on the nine human cell lines tested in Extended Data Fig. 1b, and, in all lines, live cell staining with the anti-NPM1 (FC8791) antibody demonstrated staining above isotype levels, albeit to different degrees (Fig. 2a and Extended Data Fig. 2a). While cancer cell lines are efficient at growing in vitro, over time, cultures adapt to these conditions, which may select for states that deviate from their in vivo state, where niche environments are important^30–32^. Here, we harvested leukemic marrow from four primary murine leukemia models driven by MLL-AF4 (*KMT2A-AFF1*), MLL-AF9 (*KMT2A-MLLT3*), MLL-ENL (*KMT2A-MLLT1*) and NPM1c, all in a *Flt3*^ITD/+^ background^33,34^. In all four genotypes, we observed cell surface binding with anti-NPM1 (B0556) antibody (Fig. 2b, top), demonstrating that primary murine AML also presents csNPM1. To understand whether this effect was stable ex vivo, we evaluated how csNPM1 changed upon in vitro culture of *Npm1*^cA/+^;*Flt3*^ITD/+^ AML cells. After 7 days in culture, these cells still robustly expressed csNPM1 (Extended Data Fig. 2b), although direct comparison to the in vivo data is not possible because these samples were analyzed on different days. As compared to the AML cell lines, we saw a larger distribution in the fraction and intensity of csNPM1-positive cells in primary murine AMLs. Whole-cell levels of NPM1 assessed by flow cytometry after fixation and permeabilization demonstrated similar levels and profiles across all four genotypes (Fig. 2b, bottom), suggesting that csNPM1 levels are not purely a product of total cellular abundance. The broad expression of csNPM1 in models with or without NPM1c and the higher expression in models with NPM1c (that is, OCI-AML3 and *Npm1*^cA/+^;*Flt3*^ITD/+^ murine AML) raises the question of whether both the WT and mutant forms of NPM1 can be presented on the cell surface, especially considering that NPM1c can form heterodimers with WT NPM1 (ref. ^35^). To explore this, we designed overexpression vectors encoding NPM1-WT or NPM1c with Ty1 N-terminal tags. Using an anti-NPM1 antibody, we found that the overexpression of either NPM1-WT-Ty1 or NPM1c-Ty1 further increased the levels of csNPM1 as compared to those in K562 cells bearing an empty vector (Fig. 2c). Using the anti-Ty1 antibody, we also found that both NPM1-WT-Ty1- and NPM1c-Ty1-expressing cells were detectable by flow cytometry (Fig. 2c), suggesting that both the WT and NPM1c forms are capable of reaching the cell surface. Consistently, cells expressing an empty vector had little binding when stained with the anti-Ty1 antibody (Fig. 2c). In sum, these data suggest that csNPM1 is presented in many in vitro leukemia models and in vivo primary murine AML models, regardless of NPM1 mutation status.

### Development of a monoclonal antibody to target csNPM1

The FC8791 monoclonal antibody was raised against the C-terminal region of human NPM1. This domain is highly conserved between mice, monkeys and humans (Extended Data Fig. 2c), suggesting that there is a possibility of developing a cross-species reactive antibody, which would be desirable for clinical translation. We sought to develop an antibody derived from a human immune system, thus minimizing the potential risk of anti-drug antibodies in humans^36,37^. Previously, an NPM1c mutant-specific binder was selected from a human scFv library^38^. Using this as a baseline, we constructed a mouse IgG2a antibody (mAb2) targeting NPM1 (Extended Data Fig. 2d) and evaluated its binding features. Mouse IgG2a was selected for its capacity to induce antibody-dependent cellular cytotoxicity (ADCC) and complement-dependent cytotoxicity^39^, which would enable anti-cancer immunity. First, we analyzed the signal of FC8791 and mAb2 from whole-cell lysate as well as extracted membranes, which demonstrated nearly identical banding patterns (Extended Data Fig. 2e). Next, we used mAb2 for live cell flow cytometry, and mAb2 was able to fully shift signal from OCI-AML3 cells above that of the isotype control (Fig. 2d). Evaluation of the binding pattern by confocal microscopy of OCI-AML3 cells showed that both FC8791 and mAb2 formed puncta on the cell periphery (Fig. 2e and Extended Data Fig. 2f, top). The distribution of signal was different upon fixation and permeabilization. Specifically, FC8791, which was raised against a 100% NPM1-WT peptide, displayed a highly nucleolar pattern with limited signal from the cytosol (Fig. 2e, bottom). By contrast, mAb2 had a more prominent cytosolic signal while still retaining nucleolar staining (Fig. 2e, bottom), consistent with mAb2 having some specificity for NPM1c, which is mislocalized to the cytoplasm. Co-staining live cells with mAb2 and WGA demonstrated that the mAb2 pattern was restricted to the cell surface (Extended Data Fig. 2g), and co-staining of cells with mAb2 and FC8791 demonstrated overlapping signals (Extended Data Fig. 2h), suggesting a common cell surface target. We also examined the cell surface binding of mAb2 on primary murine MLL-ENL/*Flt3*^ITD/+^ bone marrow (BM) cells, which showed poor and non-specific staining with an isotype control antibody, while mAb2 staining produced punctate, cell surface-specific clusters (Extended Data Fig. 2i) as seen on the human AML cell lines.

To validate the specificity of mAb2 using functional genetics, we generated CRISPR–Cas9 *NPM1*-knockout cells using the OCI-AML2 cell line. Upon genetic ablation of NPM1, we lost cell surface mAb2 binding, while surface expression of CD47 remained unchanged (Extended Data Fig. 2j,k), suggesting that mAb2 loss was not a non-specific finding. Finally, to further explore the csRBP clustering phenotype we observed with csNPM1 and given that we have also found glycoRNAs in proximity to other csRBP clusters^26^, we performed an RNA proximity labeling experiment on OCI-AML3 cells with mAb2. RNA from cells stained with mAb2 and then labeled with biotin-aniline revealed a high-molecular-weight biotin smear that was sensitive to RNase and sialidase (Fig. 2f), which is consistent with cell surface glycoRNA labeling^24^. Together, these data suggest that mAb2 targets csNPM1 and that this RBP is in proximity to glycoRNAs.

### csNPM1 is present on primary AML

**Fig. 3:**
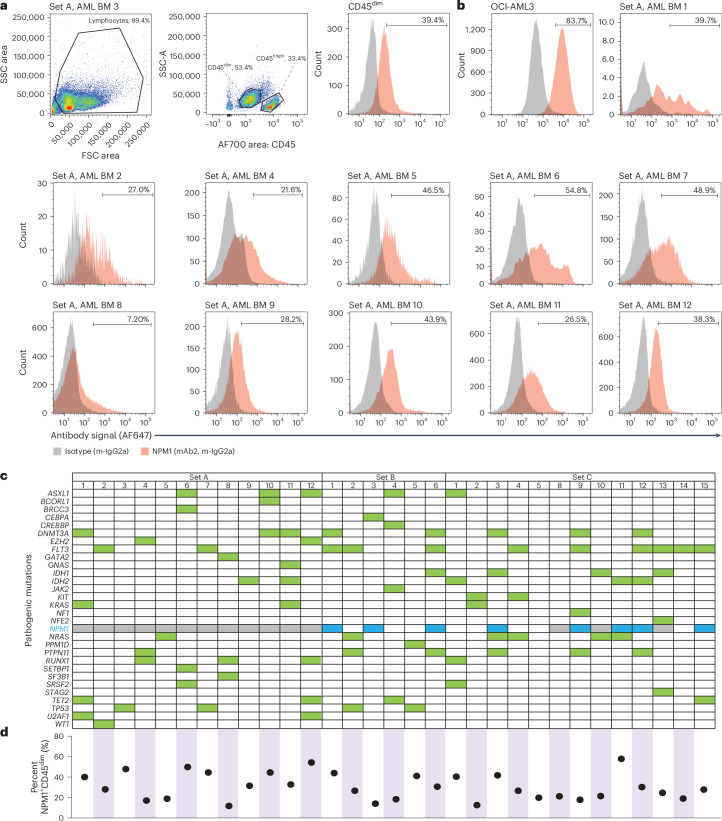
mAb2 stains primary human AML BM in a genotype-agnostic manner. **a**, Flow cytometry analysis of an AML patient BM sample (set A, 3). Gates first identify lymphocytes and then CD45^dim^ versus CD45^bright^ populations and finally the mAb2 versus isotype staining of the CD45^dim^ population in a histogram. The percent positive of each population is noted in the panels. FSC, forward scatter. **b**, Flow cytometry analysis of the OCI-AML3 cell line and 11 AML patient BM samples as analyzed in **a**. m-IgG2a, mouse-IgG2a. **c**, Pathogenic mutations identified by clinical pathology for each of the 31 AML patient BM samples analyzed in this study (across 3 sets; **a**–**c**). A green cell denotes that a mutation of that gene was identified, a gray cell denotes no testing of mutations for that gene, and blue highlights mutations in the *NPM1* gene. **d**, Dot plot of the percent of NPM1^+^ cells identified in each patient’s BM CD45^dim^ population.

**Extended Data Fig. 3:**
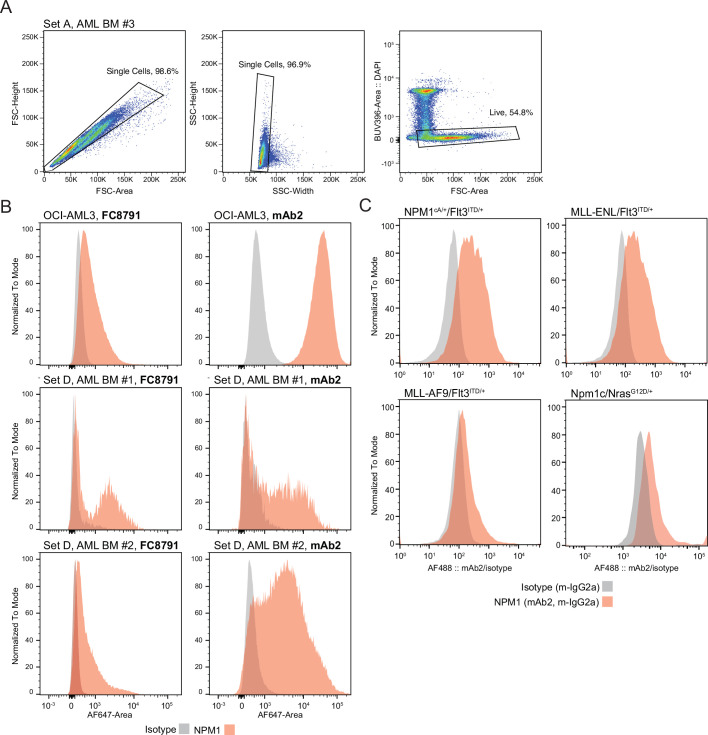
mAb2 stains multiple primary murine models of AML. **a**. Flow cytometry analysis of an AML patient bone marrow (BM) sample (Set A, #3). The three gates here demonstrate isolation of single, live cells. This gating strategy is representative of all flow cytometry in this study of patient samples. **b**. Flow cytometry analysis of OCI-AML3 cells and three AML patient bone marrow (BM) samples (Set D, #1-3). For each sample, cells were stained comparing either mouse isotype to FC8791 (left column) or mouse isotype to mAb2 (right column). **c**. Histograms of live cell flow cytometry of *NPM1*^*cA/+*^*/Flt3*^*ITD/+*^, *MLL-AF9/Flt3*^*ITD/+*^, *MLL-ENL/Flt3*^*ITD/+*^ and *Npm1c/Nras*^*G12D/+*^ primary murine cells stained with an isotype (gray) or anti-NPM1 (mAb2) (red) antibody.

Given the presence of csNPM1 on primary murine models of AML and human cell lines, we next assessed its presence on primary human AML. We obtained cryopreserved BM aspirates from healthy donors and patients with AML and performed flow cytometry using a myeloid leukemia panel (CD45, CD34, CD117, CD33, CD13, HLA-DR). Malignant blasts were coarsely identified as side scatter (SSC) low (SSC^lo^) and CD45^dim^ (Fig. 3a, middle, and Extended Data Fig. 3a). Across 12 BM aspirates (set A), including the gating example from patient 3 (Fig. 3a), the blast population generally showed a robust shift above isotype control with mAb2 (Fig. 3b). We next expanded our primary sample cohort to 31 patient samples encompassing various genotypes and immunophenotypes, including primary and secondary AMLs (that is, progressing from myelodysplastic syndrome or myeloproliferative neoplasm) (Fig. 3c and Supplementary Table 3). AML blasts consistently showed a substantially enriched csNPM1 population as compared to the isotype control (Fig. 3d). Despite the set A patients having a wide range of identified mutations through genetic analysis (Fig. 3c), all except patient 8 demonstrated clear mAb2 binding (Fig. 3b). Most profiles were unimodal, with between 21% and 54% of cells being mAb2^+^ (Fig. 3b, for example, AML BM 3, 5 and 9–12). Next we compared the relative binding efficacy of mAb2 to that of FC8791, which is a well-studied anti-NPM1 antibody (Extended Data Fig. 3b). On OCI-AML3 and two primary patient samples, we observed that both antibodies bind the malignant SSC^lo^CD45^dim^ populations, while mAb2 consistently showed higher MFI. We then examined whether mAb2 could also target csNPM1 in primary murine AML models by performing live staining followed by flow cytometry analysis, finding that mAb2 could bind these cells as well, driven by *Npm1*^cA/+^;*Flt3*^ITD/+^, MLL-AF9/*Flt3*^ITD/+^, MLL-ENL/*Flt3*^ITD/+^ and *Npm1*^c^;*Nras*^G12D/+^ (Extended Data Fig. 3c). Overall, the broadly observed and high levels of mAb2 binding on AML BM suggest that csNPM1 could serve as a genotype-agnostic marker of AML.

### csNPM1 is a myeloid-biased marker that spares healthy HSPCs

**Extended Data Fig. 4:**
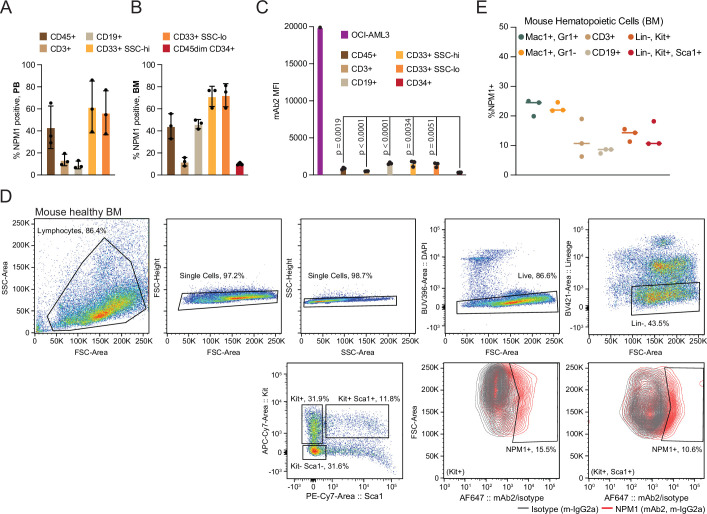
mAb2 stains the healthy hematopoietic niche poorly with no observed in vivo toxicity. **a**. Bar plot of the percent NPM1+ cells from peripheral blood (PB) samples of three independent healthy human donors. Populations analyzed include CD45+, CD3+, CD19+, CD33+ SSC-hi, CD33+ SSC-low. Error bars plotted are standard deviation (SD). **b**. Bar plot as in (A), analyzing cells from BM samples of three independent healthy human donors. The CD45dim CD34+ population is additionally analyzed in BM samples. Error bars plotted are SD. **c**. MFI analysis of mAb2 stained cells from (B) plotted on a log_10_ scale. OCI-AML3 cells are included as a positive control. Unpaired t-tests were used to calculate significance between the CD34+ cells and the other mature lineage cells, p values shown. Error bars plotted are SD. **d**. Flow cytometry analysis of a healthy mouse BM sample. The gates presented sequentially show: lymphocytes, single cells, single cells, live cells, Lin-, Kit vs. Sca1, and finally mAb2 or isotype staining on the Kit+ or Kit+ versus Sca1+ cells. This gating strategy is representative of all flow cytometry in this study of murine samples. **e**. Dot plot of the percent NPM1+ cells from BM samples of three independent healthy mouse donors. Populations analyzed include Mac1+, Gr1+; Mac1+, Gr1-; CD3+; CD19+; Lin-, Kit+; and Lin-, Kit+, Sca1+. Bars plotted are mean values.

Although mAb2 strongly stains cancer cells, avoiding healthy HSPCs will be critical to avoid the associated toxicities seen with other AML targets (that is, CD33 and CD123)^40^. To understand in detail where, and to what degree, csNPM1 is presented on healthy cells, we studied this in both human and primary mouse models. We examined this by measuring mAb2 binding on B cells (CD19), T cells (CD3), myeloid cells (CD33) and HSPCs (CD34). In both human peripheral blood (PB) and BM from healthy donors, there was minimal binding in T lymphocytes and stronger binding in CD33^+^ myeloid cells, while B lymphocytes showed higher binding in BM than in PB (Extended Data Fig. 4a,b). Both CD33^+^SCC^hi^ (neutrophils) and CD33^+^SSC^lo^ (monocytes) had ~70% mAb2 binding. To understand how this degree of positivity compares to a malignant state, we assessed the MFI for mAb2 across BM cell populations and the OCI-AML3 human AML cell line (Extended Data Fig. 4c). On average, lineage-differentiated BM populations showed tenfold less binding intensity than OCI-AML3 cells. Next, examining CD45^dim^CD34^+^ cells (HSPCs), we found an approximate tenfold reduction in binding compared to lineage cells and an approximate 100-fold lower binding than that on OCI-AML3 cells (Extended Data Fig. 4c). Thus, while a subpopulation of healthy hematopoietic cells are stained by mAb2 (percent positive), the total amount of antibody binding (MFI) is significantly less on healthy human cells than in a human AML model.

We next assessed mAb2 binding on healthy mouse hematopoietic cells. Similar to our findings with human samples, mAb2 binding was preferentially seen in BM neutrophils (MAC1^+^GR1^+^) and monocytes (MAC1^+^GR1^−^), ranging between 20% and 30% of each population (Extended Data Fig. 4d). There was ~10% binding in BM T and B cells. Again, we found low-level binding on mouse hematopoietic progenitors (lineage negative (Lin^−^)Kit^+^) and stem cells (Lin^−^Kit^+^SCA1^+^) (Extended Data Fig. 4d,e). Due to limited binding on HSPCs, we predicted that mAb2 would have a minimal impact on normal hematopoiesis.

**Fig. 4:**
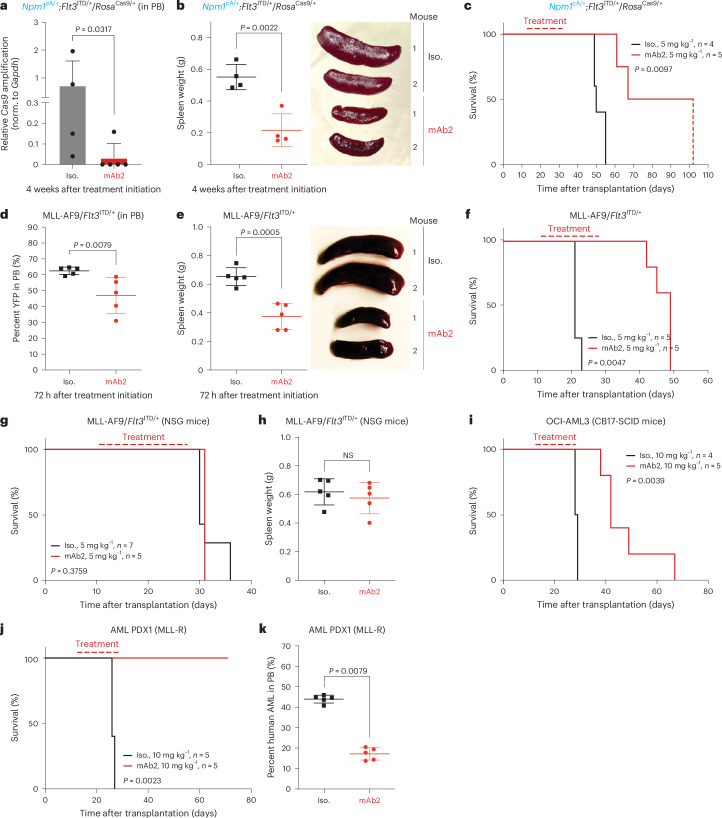
In vivo anti-AML efficacy and mechanism of killing. **a**, Real-time PCR quantification of the *Cas9* gene in PB of *Npm1*^cA/+^;*Flt3*^*I*TD/+^/*Rosa*^Cas9/+^-driven AML treated with either isotype control (Iso., *n* = 4) or mAb2 (*n* = 5) (mean ± s.d.). Statistical significance was determined by two-tailed Mann–Whitney *U*-test. Norm., normalized. **b**, Spleen weight of *Npm1*^cA/+^;*Flt3*^ITD/+^/*Rosa*^Cas9/+^ murine AML models following treatment with either the isotype control (*n* = 4) or mAb2 (*n* = 5) (mean ± s.d.). Statistical significance as in **a**. **c**, Kaplan–Meier (KM) survival after transplantation of *Npm1*^cA/+^;*Flt3*^ITD/+^/*Rosa*^Cas9/+^ treated with either the isotype control (5 mg kg^−1^, *n* = 4) or mAb2 (5 mg kg^−1^, *n* = 5). Log-rank (Mantel–Cox) test was used for survival comparisons. Dashed vertical line denotes end-of-study killing; deaths were not due to illness. **d**, Percentage of YFP^+^ MLL-AF9/*Flt3*^ITD/+^ cells in PB 72 h after treatment with either the isotype control (5 mg kg^−1^) or mAb2 (5 mg kg^−1^) (*n* = 5, mean ± s.d.). Statistical significance as in **a**. **e**, Spleen weight of MLL-AF9/*Flt3*^ITD/+^ mice 72 h after treatment with either the isotype control (5 mg kg^−1^) or mAb2 (5 mg kg^−1^) (*n* = 5, mean ± s.d.). Statistical significance as in **a**. **f**, KM survival after transplantation of MLL-AF9/*Flt3*^ITD/+^ cells in PB 72 h after treatment with either the isotype control (5 mg kg^−1^, *n* = 5) or mAb2 (5 mg kg^−1^, *n* = 5). Survival comparisons as in **c**. **g**, KM survival after transplantation of MLL-AF9/*Flt3*^ITD/+^ in NSG mice treated with either the isotype control (5 mg kg^−1^, *n* = 7) or mAb2 (5 mg kg^−1^, *n* = 5). Survival comparisons as in **c**. **h**, Spleen weight of NSG mice after transplantation of MLL-AF9/*Flt3*^ITD/+^ cells, treated with either the isotype control (5 mg kg^−1^) or mAb2 (5 mg kg^−1^) (*n* = 5, mean ± s.d.). Significance as in **a**. **i**, KM survival after transplantation of OCI-AML3 cells in CB17-SCID mice treated with either the isotype control (10 mg kg^−1^, *n* = 4) or mAb2 (10 mg kg^−1^, *n* = 5). Survival comparisons as in **c**. **j**, KM survival after transplantation of an AML PDX (MLL-R) in CB17-SCID mice treated with either the isotype control (10 mg kg^−1^, *n* = 5) or mAb2 (10 mg kg^−1^, *n* = 5). Survival comparisons as in **c**. **k**, Percentage of human myeloid cells in PB after transplantation of an AML PDX (MLL-R) in CB17-SCID mice on day 24 after transplantation and after 2 treatments of either isotype control (10 mg kg^−1^) or mAb2 (10 mg kg^−1^) (*n* = 5, mean ± s.d.). Significance as in **a**.

**Extended Data Fig. 5:**
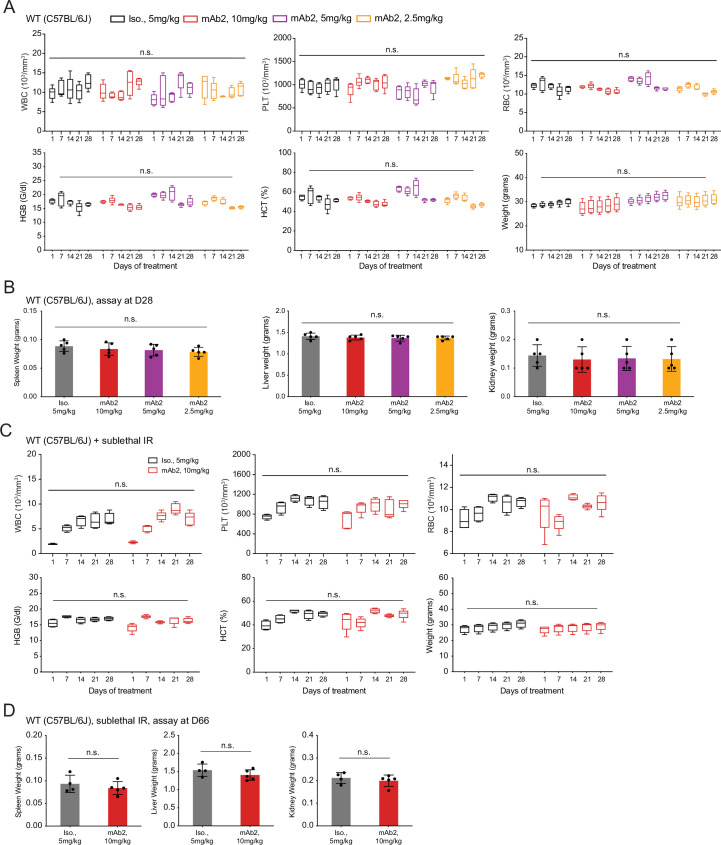
Poor presentation of csNPM1 on normal mouse bone marrow. **a**. Blood count results and total body weight from an animal experiment with WT C57BL/6J mice following 4 weekly treatments with either 5mg/kg isotype control (Iso.) or 2.5mg/kg, 5 mg/kg, and 10 mg/kg of mAb2 (n=5 mice used in each group). Statistical significance was determined by two-tailed Mann–Whitney U test and box plots showing median, IQR, and extremes. Whiskers extend from minimum to maximum values in all the box plots presented. **b**. Spleen, liver, and kidney weight from WT C57BL/6J mice following 4 weekly treatments with either Iso. or 2.5, 5, and 10 mg/kg of mAb2 (n=5 mice used in each group) (mean ± SD). Statistical significance was determined by two-tailed Mann–Whitney U test. **c**. Blood count results and total body weight from an animal experiment with sub-lethally irradiated WT C57BL/6J mice following 4 weekly treatments with either 5mg/kg Iso. or 10 mg/kg of mAb2 (n=5 mice used in each group). Statistical significance was determined by two-tailed Mann–Whitney U test and box plots showing median, IQR, and extremes. Whiskers extend from minimum to maximum values in all the box plots presented. **d**. Spleen, liver, and kidney weight from sub-lethally irradiated WT C57BL/6J mice following 4 weekly treatments with either 5mg/kg Iso. or 10 mg/kg of mAb2 (n=5 mice used in each group) (mean ± SD). Statistical significance was determined by two-tailed Mann–Whitney U test.

To examine the potential toxicity that mAb2 treatment might have in vivo, we used WT (C57BL/6J) mice and injected them intraperitoneally (i.p.) with three different doses (2.5, 5 and 10 mg kg^−1^) of mAb2 or an isotype control (5 mg kg^−1^). No significant differences were observed in PB counts or the total body weight of mice after 4 weekly treatments at any given dose (Extended Data Fig. 5a). At the end of treatment, no significant weight differences were observed in the spleens, livers or kidneys (Extended Data Fig. 5b). We next examined the impact of mAb2 treatment in sublethally irradiated mice. After weekly injections of 10 mg kg^−1^ of mAb2 or 5 mg kg^−1^ of isotype control, there were no differences in blood counts or total body weight between treatment arms after 4 weeks (Extended Data Fig. 5c). These mice were kept for 5 additional weeks after the final treatment, and there were still no detectable health issues or tissue weight changes observed (Extended Data Fig. 5d). Overall, these data suggest that the mAb2 antibody is well tolerated and shows no transient or lasting toxicity on normal hematopoiesis in vivo.

### In vivo efficacy of mAb2 in mouse AML models

**Extended Data Fig. 6:**
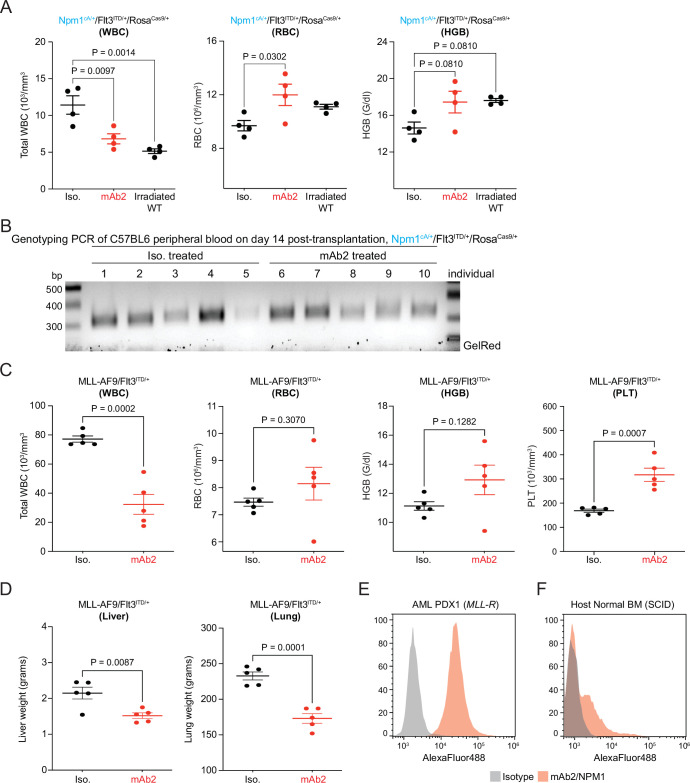
mAb2 treatment reduces leukemic burden in mice. **a**. Blood count results after transplantation of *Npm1*^*cA/+*^*/Flt3*^*ITD/+*^*/Rosa*^*Cas9/+*^ in sub-lethally irradiated WT C57BL/6J mice treated with either Iso. (5 mg/kg) or mAb2 (5 mg/kg) (n=4, mean ± SD. Statistical significance was determined by a two-tailed Mann–Whitney U test. **b**. DNA gel of genotyping PCR of the PB on day 14 post transplantation of *Npm1*^*cA/+*^*/Flt3*^*ITD/+*^*/Rosa*^*Cas9/+*^ in sub-lethally irradiated WT C57BL/6J mice treated with either Iso. (5 mg/kg) or mAb2 (5 mg/kg). **c**. Blood count results after transplantation of *MLL-AF9/Flt3*^*ITD/+*^ in sub-lethally irradiated WT C57BL/6J mice treated with either Iso. (5 mg/kg) or mAb2 (5 mg/kg) (n=5, mean ± SD). Statistical significance was determined by two-tailed Mann–Whitney U. **d**. Liver and lung weight of *MLL-AF9/Flt3*^*ITD/+*^ murine AML mice treated with either Iso. (5 mg/kg) or mAb2 (5 mg/kg) (n=5, mean ± SD). Statistical significance was determined by Mann–Whitney two-tailed U test. **e**. Histogram of live cell flow cytometry on BM-derived AML PDX (*MLL-R*) stained with Iso. (gray) and mAb2 (red). **f**. Histogram of live cell flow cytometry on host normal BM (SCID) from the PDX shown in (D).

To establish whether mAb2 has anti-leukemic efficacy, we first used a primary mouse AML model driven by *Npm1*^cA/+^;*Flt3*^ITD/+^/*Rosa*^Cas9/+^. This model possesses the highest levels of csNPM1 among the primary murine models tested (Fig. 2b and Extended Data Fig. 3c). We used sublethally irradiated, immunocompetent mice, which were injected with primary murine *Npm1*^cA/+^;*Flt3*^ITD/+^/*Rosa*^Cas9/+^ AML cells. After AML engraftment, mice were treated weekly with either isotype control or mAb2 at 5 mg kg^−1^. Examining the PB 4 weeks after treatment, mice treated with mAb2 had significantly lower levels of the *Cas9* transgene than those given the isotype control (Fig. 4a). Moreover, we observed significantly lower white blood cell counts in mice treated with mAb2 than in those treated with the isotype control, indicating decreased leukemic burden. With improved leukemia control, there was hematopoietic recovery, as shown by white blood cell counts, red blood cells and hemoglobin returning to irradiation-only control levels (Extended Data Fig. 6a,b). Because splenomegaly is one of the key indicators of AML engraftment and expansion in vivo, we examined spleen sizes and found that the mAb2-treated cohort had significantly smaller spleens than the isotype-treated cohort (Fig. 4b). Finally, examination of the overall survival demonstrated that weekly i.p. treatment with mAb2 significantly prolonged survival when compared to the isotype-treated cohort (Fig. 4c). Overall, our results show that mAb2 treatment impaired leukemic engraftment and expansion in a primary murine *Npm1*^cA/+^;*Flt3*^ITD/+^/*Rosa*^Cas9/+^ AML model while significantly prolonging survival.

Based on mAb2’s efficacy in high-csNPM1 models, we next assessed its in vivo efficacy in AML models with lower levels of csNPM1. We used the primary murine MLL-AF9/*Flt3*^ITD/+^ AML model, which showed an intermediate amount of mAb2 binding (Extended Data Fig. 3c). These cells, which contain a yellow fluorescent protein (YFP) marker, were transplanted and treated as above. We observed that the AML burden (YFP^+^) in PB was significantly lower in the mAb2-treated cohort than in the cohort treated with the isotype control after just 72 h of treatment (Fig. 4d). Total blood counts further showed that markers of leukemia were reduced in the treated cohort, while again red blood cells, hemoglobin and platelets were all increased, suggesting improved hematopoiesis (Extended Data Fig. 6c). Notably, the leukemia-infiltrated spleen, liver and lungs were significantly smaller in the mAb2-treated cohort than in the cohort treated with the isotype control, suggesting strong anti-leukemic efficacy in those tissues (Fig. 4e and Extended Data Fig. 6d). Treatment with mAb2 significantly prolonged survival (Fig. 4f), indicating that mAb2 can also effectively target AML with intermediate csNPM1 levels.

To establish the killing mechanism of mAb2, we transplanted MLL-AF9/*Flt3*^ITD/+^ AML into immunocompromised mice (NOD severe combined immunodeficiency disease (SCID) gamma (NSG)), which lack natural killer cells to participate in Fc-mediated ADCC and also have an impaired complement system. In NSG mice, we did not observe any differences in the overall survival or the spleen size of mice treated with mAb2 compared to the isotype control (Fig. 4g,h). These findings suggest that mAb2’s mechanism of action is dependent on ADCC and/or complement-dependent cytotoxicity. To further investigate our findings, we used CB17-SCID mice, which are deficient in B and T cells but have intact innate immunity, including complement^41^. CB17-SCID mice harboring human OCI-AML3 cells had significantly improved survival in the mAb2-treated cohort (Fig. 4i), solidifying that the anti-leukemic effect we observe is immune mediated.

Finally, to assess whether mAb2 has the potential to target human AML patient cells in vivo, we performed flow cytometry analysis to examine csNPM1 levels in a patient-derived xenograft (PDX) model driven by an MLL rearrangement (MLL-R), which is seen in 40% of pediatric leukemia and 5–12% of adult AML^42^. We found high csNPM1 levels on the engrafted PDX model, whereas the host mouse BM had significantly lower csNPM1 levels (Extended Data Fig. 6e,f). Finally, mAb2 treatment of CB17-SCID mice bearing this PDX led to significant survival prolongation (Fig. 4j) and reduced PDX engraftment (Fig. 4k). These results suggest that mAb2 is highly efficacious in vivo and in the context of clinically relevant human AML.

### mAb2 targets leukemic stem cells

**Fig. 5:**
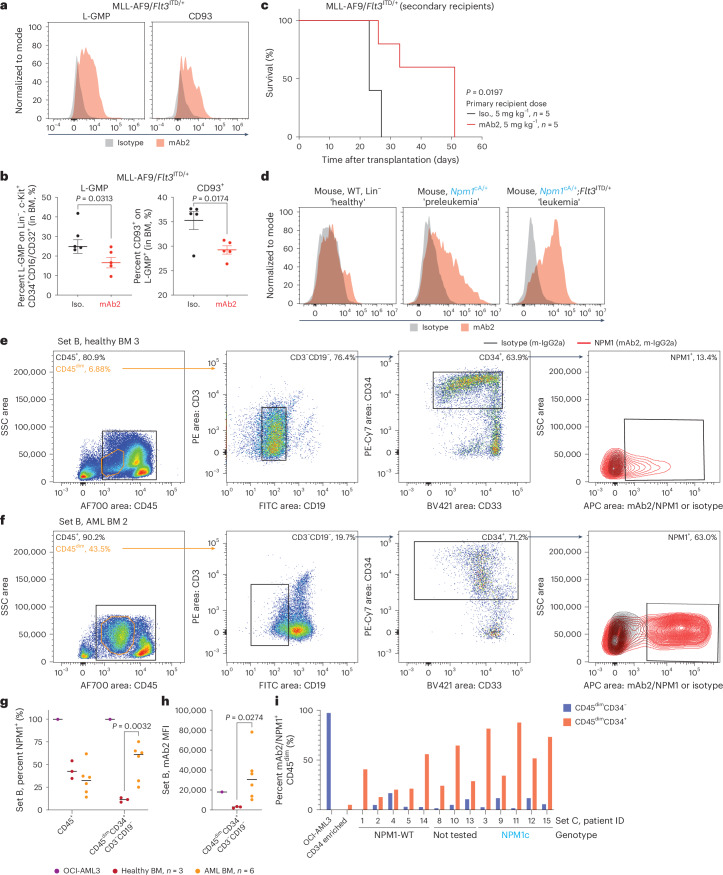
mAb2 treatment targets key LSCs. **a**, Histogram of live cell flow cytometry from primary murine MLL-AF9/*Flt3*^ITD/+^ L-GMP^+^ (left) and CD93^+^ (right) LSC populations stained with an isotype control (gray) or mAb2 (red) antibody. **b**, Percentage of L-GMP^+^ (left) or CD93^+^ (right) cells in BM of the MLL-AF9/*Flt3*^ITD/+^ model treated with either isotype control (*n* = 5) or mAb2 (*n* = 5). Statistical significance was determined by two-tailed Mann–Whitney *U*-test, and bars show median ± s.d. **c**, KM survival after retransplantation of cells isolated from primary recipients transplanted with MLL-AF9/*Flt3*^ITD/+^ cells and treated with either isotype control (*n* = 5) or mAb2 (*n* = 5). The log-rank (Mantel–Cox) test was used for survival comparisons. **d**, Histograms of live cell flow cytometry using WT, *NPM1*^cA/+^ and *NPM1*^cA/+^;*Flt3*^ITD/+^ primary murine BM HSPCs (Lin^−^) stained with an isotype control (gray) or mAb2 (orange) antibody. **e**, Flow cytometry analysis of a healthy donor BM sample (set B, 3). Gates first identify CD45^dim^ and then Lin^−^ and then CD34^+^ and finally display mAb2 or isotype control antibody binding. The percent positive of each population is noted in the panels. **f**, Flow cytometry analysis as in **a** of an AML patient BM sample (set B, 2). **g**, Dot plot of the percent of NPM1^+^ cells from OCI-AML3 cells and set B healthy donor and patient BM samples. Whole CD45^+^ versus CD45^dim^CD3^−^CD19^−^CD34^+^ cells are analyzed separately. Statistical significance was determined by an unpaired *t*-test; *P* value is noted. Bars displayed are median values. **h**, MFI analysis of mAb2-stained cells from **c**. Unpaired *t*-tests were used to calculate significance between the healthy and AML BM samples. Statistical significance was determined by an unpaired *t*-test; *P* value is shown. Bars displayed are median values. **i**, Bar plot of the percent of NPM1^+^ cells from OCI-AML3 cells, human CD34^+^ enriched healthy cells or 13 BM samples from AML patients (set C). Data are stratified by CD34 positive (red) or CD34 negative (blue), and the NPM1c status is noted below each sample. Source data

It has been reported that IL-7R^−^Lin^−^Sca-1^−^c-Kit^+^CD34^+^FcγRII/III^+^ GMP-like leukemic cells (L-GMP) and CD93^+^ subpopulations are linked to the generation and maintenance of AMLs driven by MLL^43,44^. Using the primary murine MLL-AF9/*Flt3*^ITD/+^ AML model (treatment as in Fig. 4d), we detected considerable csNPM1 levels on the surface of L-GMP^+^ and CD93^+^ populations compared to the isotype control (Fig. 5a). Additionally, there was a significant reduction of L-GMP^+^ and CD93^+^ subpopulations in the mAb2-treated cohort when compared to the isotype control-treated mice (Fig. 5b). To show that mAb2 treatment leads to functional impairment of leukemic stem cells, we performed secondary transplantations using the engrafted BM from leukemic mice treated with either mAb2 or isotype control (treated as in Fig. 4d). Following secondary transplantation, mice were left untreated, and we observed that secondary recipients from the mAb2 cohort showed improved overall survival as compared to the isotype control (Fig. 5c). These results indicate that mAb2 treatment targets AML stem cell- or leukemia-propagating compartments.

Furthermore, to assess the potential of csNPM1 as a marker of leukemia initiation and progression, we examined mAb2 surface staining of isogenic primary murine BM HSPCs (Lin^−^) from WT, *Npm1*^cA/+^ or *Npm1*^cA/+^;*Flt3*^ITD/+^ mice. In this model, *Npm1*^cA/+^ has a preleukemic phenotype, while *Npm1*^cA/+^;*Flt3*^ITD/+^ mice have overt leukemia. There was a clear ‘stage-dependent’ increase in mAb2 surface binding where *Npm1*^cA/+^;*Flt3*^ITD/+^ cells had evidently more csNPM1 than the isogenic *Npm1*^cA/+^ preleukemic cells or the WT BM, which had little to no surface binding (Fig. 5d). In summary, these findings suggest that csNPM1 may be preferentially expressed in the leukemia-initiating cell fraction in murine models of leukemia.

To explore csNPM1 binding on the HSPC population from primary patient leukemia samples, we coarsely defined HSPCs as CD45^dim^CD3^−^CD19^−^CD34^+^ cells^45^. As expected, this immature population was in low abundance in healthy BM (Fig. 5e, set B) and poorly stained with mAb2 over the isotype signal (13.4% positive; Fig. 5e). Examining a BM aspirate from a patient with AML demonstrated a markedly expanded CD45^dim^ population, representing malignant blasts. This patient’s blasts had aberrant CD19 expression, which is a relatively common phenomenon in AML^46^. Within this sample, there was a minor population of CD19^−^CD3^−^CD34^+^ HSPCs, which were demonstrably better bound by mAb2, both in percent positive (63%) and fluorescent intensity (Fig. 5f).

**Extended Data Fig. 7:**
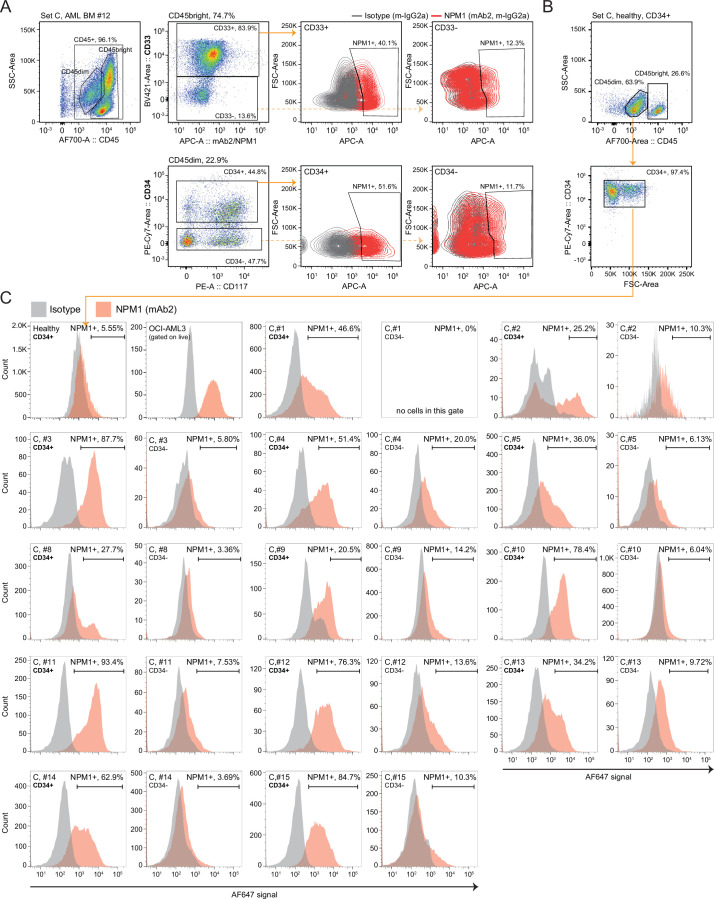
mAb2 binding to primary AML bone marrow and leukemic stem cells. **a**. Flow cytometric gating strategy for deriving CD45dim blast cells and CD45 bright mature cells, and NPM1 expression within these subsets. **b**. Gating strategy showing cell purity from CD34 enriched healthy control. **c**. Histograms of live flow cytometry from CD34-enriched healthy control, OCI-AML3, and 13 AML bone marrow aspirates. When measurable, NPM1 positivity is shown within the CD45dim CD34+ and CD45dim CD34- populations for each AML bone marrow aspirate.

To understand how this pattern appears across AML patient sample set B (three healthy and six AML BM aspirates), we first looked at all CD45^+^ cells, where 34–54% of healthy and 14–61% of AMLs were mAb2^+^, with the reference OCI-AML3 being 100% positive (Fig. 5g). However, when next considering the CD45^dim^CD34^+^CD3^−^CD19^−^ stem-like population, only 8–13% of healthy samples but 25–75% of AML samples were mAb2^+^ (Fig. 5g). Motivated by the intensity difference seen between Fig. 3a,b, we next quantified the MFI of mAb2 binding on CD45^dim^CD34^+^CD3^−^CD19^−^ HSPCs. HSPC populations from AML samples had significantly higher MFI values of mAb2 binding than the homogeneously low MFI values observed on healthy HSPCs (Fig. 5h). This set of healthy and patient BM samples suggests that, by both fraction bound and intensity bound, mAb2 selectively binds csNPM1 on the surface of primitive malignant cells. We expanded this type of analysis to examine the 13 patient samples in set C (Fig. 5i and Extended Data Fig. 7a–c). NPM1c leukemic blasts are generally CD34 negative, unlike most other AML subtypes. However, studies characterizing the minor fraction of CD34^+^ cells from NPM1c samples show that mutated NPM1 protein can also be found in this subset. Additionally, when CD34^+^ cells from NPM1c samples are transplanted, they can reconstitute a CD34^−^ leukemia, thus suggesting that CD34^+^ cells from NPM1c are leukemia-initiating cells^47^. First looking at one patient with AML (set C, AML BM 12), we observed that csNPM1 expression among CD45^dim^ cells was predominantly within the CD34^+^ fraction (Extended Data Fig. 7a). Analyzing the other 12 patients as well as CD34^+^ enriched healthy donor cells showed that csNPM1 was upregulated in the CD34^+^ subset of all AML samples (Extended Data Fig. 7b,c). The CD45^dim^CD34^+^ fraction from NPM1c samples had the most csNPM1 signal as compared to CD34^−^ blasts. Overall, in human samples, there is a clear preferential binding for csNPM1 in the HSPC population of malignant marrow that is not present in healthy marrow. Additional studies will be needed to assess whether this binding is preferential to bona fide human leukemic stem cells.

### mAb2 enables in vivo targeting of solid tumors

**Fig. 6:**
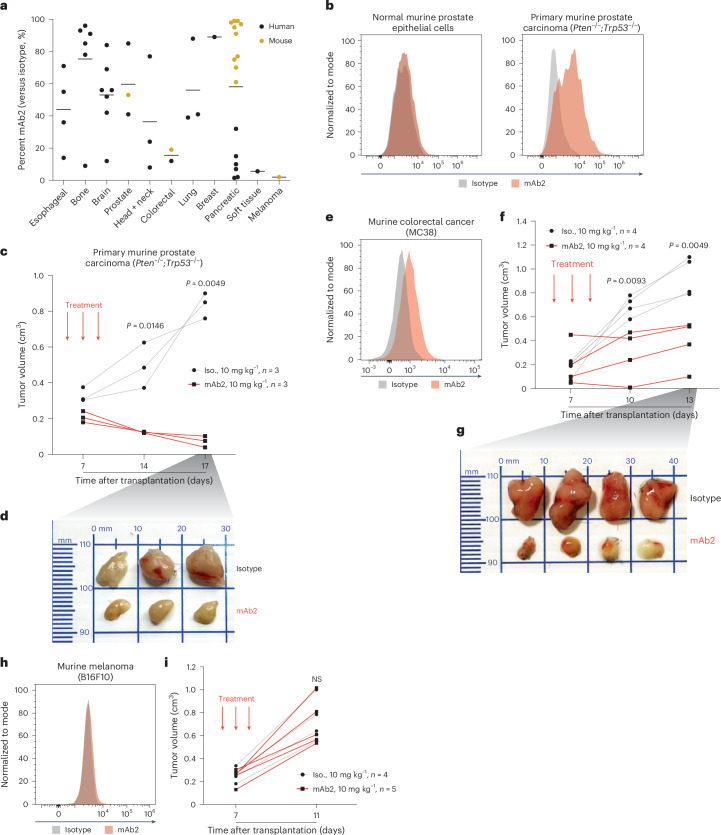
mAb2 stains multiple solid cancer models and impairs in vivo tumor growth. **a**, Percent NPM1^+^ cells from flow cytometric analysis of 47 human and mouse solid cancer models from various tissue origins (Supplementary Table 4). Bars show mean per tissue. **b**, Histogram of live cell flow cytometry from primary, healthy murine prostate epithelial cells (left) or primary murine *Pten*^−/−^;*Trp53*^−/−^ prostate carcinoma cells (right) stained with an isotype control (gray) or mAb2 (orange) antibody. **c**, Tumor volume of the primary murine *Pten*^−/−^;*Trp53*^−/−^ prostate carcinoma model following treatment with either isotype control (*n* = 3) or mAb2 (*n* = 3) (mean ± s.d.). Syngeneic tumors were engrafted subcutaneously, and treatment began after development of palpable tumors. Red arrows indicate the relevant time points of treatment. Statistical significance was determined by two-tailed Mann–Whitney *U*-test. **d**, Primary murine *Pten*^−/−^;*Trp53*^−/−^ prostate carcinoma tumors from the animal cohorts shown in **c**, treated with either the isotype control (top) or mAb2 (bottom) and dissected on day 17 after transplantation. **e**, Histogram of live cell flow cytometry from the mouse colorectal cancer model MC38 stained with an isotype control (gray) or mAb2 (orange) antibody. **f**, Tumor volume of the mouse colorectal cancer model MC38 following treatment with either isotype control (*n* = 4) or mAb2 (*n* = 4) (mean ± s.d.). Syngeneic tumors were engrafted subcutaneously, and treatment began after development of palpable tumors. Red arrows indicate the relevant time points of treatment. Statistical significance was determined by two-tailed Mann–Whitney *U*-test. **g**, MC38 tumors from the animal cohorts shown in **f**, treated with either the isotype control (top) or mAb2 (bottom) and dissected on day 13 after transplantation. **h**, Histogram of live cell flow cytometry from the mouse melanoma cancer model B16F10 stained with an isotype control (gray) or mAb2 (orange) antibody. **i**, Tumor volume of the mouse melanoma cancer model B16F10 following treatment with either isotype control (*n* = 4) or mAb2 (*n* = 5) (mean ± s.d.). Syngeneic tumors were engrafted subcutaneously, and treatment began after development of palpable tumors. Red arrows indicate the relevant time points of treatment. Statistical significance was determined by two-tailed Mann–Whitney *U*-test. Source data

Given the genotype-agnostic binding of mAb2 across a diverse set of AML models and primary samples, we hypothesized that csNPM1 could also mark solid tumors. To assess this, we measured mAb2 binding across 47 human and mouse solid tumor models from various tissue origins using flow cytometry (Fig. 6a and Supplementary Table 4). We saw varying degrees of mAb2 binding in multiple tissue types. There was no binding in B16F10 murine melanoma and little binding in multiple models of human pancreatic cancer. Given the binding on solid tumors, we next sought to assess whether we could achieve in vivo efficacy with mAb2. We turned to a syngeneic model of prostate cancer, in which primary normal murine prostate epithelial cells had little to no mAb2 binding while primary murine prostate carcinoma cells (*Pten*^−/−^;*Trp53*^−/−^) had clear staining above isotype (Fig. 6b). After engrafting syngeneic tumors in C57BL/6 mice, animals were treated on days 5, 7 and 9 with 10 mg kg^−1^ of mAb2 or isotype control. We found that mAb2 treatment reduced the tumor volume, while isotype-treated animals had significantly larger tumors at 14 and 17 days after engraftment (Fig. 6c,d). To extend this observation beyond prostate carcinoma, we also evaluated the activity of mAb2 against a murine colorectal cancer model (MC38) that is also bound by mAb2 (Fig. 6e). In this syngeneic model, we also observed significant reduction in tumor volume at 10 and 13 days after transplantation in C57BL/6 mice (Fig. 6f,g). Finally, to establish the specificity of this effect, we examined the activity of mAb2 on the murine melanoma line B16F10, which has no significant mAb2 binding (Fig. 6a,h). As expected, treatment of B16F10-transplanted syngeneic mice with mAb2 has no impact on tumor volume when compared to isotype-treated controls (Fig. 6i). These data suggest that mAb2 induces anti-tumor activity in vivo in csNPM1-positive solid tumors.

## Discussion

Here, we show that NPM1, a frequently mutated protein in AML and an often dysregulated protein in malignancy, can be found on the cell surface. Because csRBPs such as NPM1 lack a transmembrane domain and glycosylphosphatidylinositol-linked anchors, we focused on the molecular organization of csNPM1 by combining mass spectrometry (MS) and SR microscopy methods. Cell surface proximity labeling defined many other csRBPs and glycoRNAs within the molecular neighborhoods of csNPM1, consistent with our recent observation of csRBP clusters (for example, DDX21, hnRNP-U, etc.) on other cell types^26^. Similarly, the SR reconstructions of csNPM1 on AML cells are highly similar to those of DDX21 and hnRNP-U on adherent cell lines^26^; such a non-random, structured organization suggests the existence of an underlying regulatory mechanism guiding the nanoscale arrangement of these clusters.

Although this study does not fully elucidate the mechanism that drives the surface expression of csNPM1, we do identify that csNPM1 from membrane fractions is bound by sialic acid-specific lectins. Given the predicted *O*-linked glycosylation sites in NPM1, these findings would suggest that NPM1 can be glycosylated. Protein glycosylation is not only critical to appropriate folding, but it can dictate trafficking, which may in turn be indispensable for NPM1’s cell surface localization. Interestingly, others have identified that cell surface nucleolin is glycosylated, and this modification is important for its localization^48^. There is much to be learned about the mechanisms that govern how csRBPs are glycosylated and how this might relate to malignancy.

Aberrant protein expression and localization is a common feature of malignancies. Recent work from our groups has shown that the glycoRNA–csRBP clusters can serve as regulators of cell-penetrating peptide entry^26^, while others have shown that csRBPs can bind viruses and mediate ligand internalization^49^. Given these findings and the commonality of csNPM1 in multiple models of malignancy shown here, this suggests that csNPM1 and its molecular neighborhood on the cell surface may play a functional role in tumorigenesis. Future work enabled by new tools to manipulate csRBPs directly could allow for a direct understanding of csNPM1’s (and that of other csRBPs) impact on cellular state.

In this study, we focused on csNPM1 as a target for opsonization and immune-mediated destruction of target cells. This approach, if successfully translated to humans, offers substantial advancements in existing anti-cancer therapies. First, csNPM1 expression was mutation agnostic, which enables its potential use in a multitude of patients. Current advancements in AML therapy have used small molecules that work on a subset of patients based on their driver mutations^50,51^. Second, other approved anti-AML therapies that are not mutation specific work by inducing DNA damage, which can cause off-target toxicities^52^. Within the healthy hematopoietic system, csNPM1 has limited expression and importantly is not present on healthy HSCs. Whereas most approved therapies drive life-threatening cytopenias by also affecting normal hematopoiesis^53^, we do not see this in mouse models. Upon mAb2 treatment of mice with AML, we saw improvement in blood counts, which indicate simultaneous eradication of leukemia and unperturbed normal hematopoiesis.

Furthermore, there were no observable weight changes in mice to suggest broader toxicity, which is reassuring, as NPM1 is well conserved between humans and mice. Ultimately, non-human primate and human studies will be needed to assess whether csNPM1 is expressed on vital non-hematopoietic tissue. If it was expressed on healthy tissues, one interesting prospect is to develop antibodies against mutant NPM1, as we observed both WT and mutant forms on NPM1-mutant models. By targeting a mutant form with an antibody, one could limit off-target effects further. Interestingly, given that there are other RBPs mutated in AML^54^, if they are also found to be on the cell surface, they could represent novel targets.

Finally, an important goal in AML therapy development has been to target leukemic stem cells^55^. In our mouse models, we indeed see that csNPM1 is a marker of LSCs, which may in part explain the significant survival benefit offered by anti-csNPM1 therapy. Although our study is not powered to assess human LSCs, we do see increased anti-NPM1 expression in the CD34^+^ fraction of NPM1c AML marrow, which is notable for having CD34^−^ blasts. This would suggest that there is a primitive population that has csNPM1 in these samples, which we do not observe in healthy BM tissue. Phenotypically, LSCs are defined by their ability to initiate leukemia in limiting dilution transplantation experiments^55^. Therefore, a focus of future efforts will be to characterize csNPM1^+^ leukemic cells in transplantation models that have been created to foster human leukemogenesis. LSCs are better characterized in mouse leukemia models^43,44^, and, as we illustrated in this study, csNPM1 is upregulated in well-defined and clinically relevant murine LSCs.

The finding of csNPM1 prompts a need to reevaluate cell surface proteomics to ensure that non-canonical findings are studied, rather than discarded as noise. With multiple examples now of cancer-specific csRBPs (csNPM1, cs-U5 snRNP200 (ref. ^19^), etc.), there is a clear need to better investigate their biology in other normal and abnormal contexts. Beyond monoclonal antibodies, these proteins could serve as optimal targets for antibody–drug conjugates and chimeric antigen receptor cell products, thus bringing novel therapies to the patients who need them most.

## Methods

### Cell culture

All cells were cultured at 5% CO_2_ and 37 °C. Suspension cell lines including OCI-AML2, OCI-AML3, Nalm6, MOLM-13, Jurkat, Jeko1, SupT1, K562, HL-60 and Kasumi-1 were maintained by centrifuging cells, removing the medium and resuspending cells in fresh complete medium. Suspension cell cultures were split when cell density reached 2 million cells per ml. Culture medium used was 1× RPMI 1640 base medium (Thermo Fisher Scientific) with 1% penicillin–streptomycin (Pen/Strep) and 10% heat-inactivated FBS (Thermo Fisher Scientific). OCI-AML2 and OCI-AML3 cells were cultured in MEM α medium (Fisher Scientific) supplemented with 20% FBS (Fisher Scientific) and 1% Pen/Strep. A549, MC38, A673, SH-SY5Y, H4, U251, MDA-MB-231, B16F10 and primary mouse PDAC1-10 (ref. ^56^) cells were cultured in DMEM medium (Fisher Scientific) supplemented with 10% FBS (Fisher Scientific) and 1% Pen/Strep. LA-N-5 cells were cultured in DMEM (Fisher Scientific) supplemented with 20% FBS (Fisher Scientific) and 1% Pen/Strep. KNS-42 cells were cultured in DMEM (Fisher Scientific) supplemented with 10% FBS (Fisher Scientific), 1% sodium pyruvate and 1% Pen/Strep. UT-SSC-42B cells were cultured in DMEM (Fisher Scientific) supplemented with 10% FBS (Fisher Scientific), 1% NEAA and 1% Pen/Strep. Calu-1, HCT-116, MHH-ES-1 and U2OS cells were cultured in McCoy’s 5A medium (Fisher Scientific) supplemented with 10% FBS (Fisher Scientific) and 1% Pen/Strep. SaOS cells were cultured in McCoy’s 5A (Fisher Scientific) supplemented with 15% FBS (Fisher Scientific) and 1% Pen/Strep. HOS, MG-G3, FADU, DETROIT, HT1080 and HPAF-II cells were cultured in MEM medium (Fisher Scientific) supplemented with 10% FBS (Fisher Scientific) and 1% Pen/Strep. NCI-H520, KYSE-30, KYSE-140, SiMa, PFSK1, PC3, 22Rv1, ASPC1, BXPC3, SU86.86 and YAPC cells were cultured in RPMI 1640 (Thermo Fisher Scientific) supplemented with 10% FBS (Fisher Scientific) and 1% Pen/Strep. OE21 and OE33 cells were cultured in RPMI 1640 (Thermo Fisher Scientific) supplemented with 10% FBS (Fisher Scientific), 2% l-glutamine (Thermo Fisher Scientific) and 1% Pen/Strep. All the primary murine AML cells were cultured in X-VIVO 20 medium with gentamicin and PR1 (Lonza) supplemented with 5% FBS, recombinant murine IL-3 (10 ng ml^−1^, PeproTech), IL-6 (10 ng ml^−1^, PeproTech) and SCF (50 ng ml^−1^, PeproTech) and 1% penicillin–streptomycin–glutamine (Gibco). Cell lines were acquired from ATCC and the Sanger Institute Cancer Cell Collection unless otherwise noted. A full list of the cell lines’ source is provided in the Reporting summary. Cell cultures were periodically checked for *Mycoplasma* and maintained as *Mycoplasma* negative. Human cell lines employed were either not listed in the cross-contaminated or misidentified cell line database curated by the International Cell Line Authentication Committee or were previously verified by karyotyping.

### Ex vivo culture of murine primary leukemia

*Flt3*^ITD/+^ (*Flt3* internal tandem duplication) mice were kindly provided by G. Gilliland (Harvard Medical School, USA) and crossed with *Rosa26*^Cas9/+^ mice. Freshly isolated BM from 6–10-week-old female *Flt3*^ITD/+^;*Rosa26*^Cas9/+^ or moribund *Npm1*^fl−cA/+^;*Flt3*^ITD/+^;*Rosa26*^Cas9/+^, *Npm1*^fl−cA/+^;*Nras*^G12D/+^ mice were used. BM cells were exposed to erythrocyte lysis (BD Pharm Lyse, BD Biosciences), followed by magnetic bead selection of Lin^−^ cells using the Lineage Cell Depletion Kit (Miltenyi Biotec) according to the manufacturer’s instructions. Lin^−^ cells were cultured in X-VIVO 20 (Lonza) supplemented with 5% FBS (Life Technologies), 10 ng ml^−1^ IL-3 (PeproTech), 10 ng ml^−1^ IL-6 (PeproTech) and 50 ng ml^−1^ SCF (PeproTech) and 1% penicillin–streptomycin–glutamine. The retroviral constructs pMSCV-MLL-AF9-IRES-YFP and pMSCV-MLL-ENL-IRES-Neo were used with the package plasmid psi-Eco to produce retrovirus. Next, 293T cells (Life Technologies) were cultured and prepared for transduction in 10-cm plates. For virus production, 5 μg of the above plasmids and 5 μg of the psi-Eco packaging vector were transfected dropwise into the 293T cells using 47.5 μl TransIT-LT1 (Mirus) and 600 μl Opti-MEM (Invitrogen). The resulting viral supernatant was collected as previously described. Transduction of primary *Flt3*^ITD/+^;*Rosa26*^Cas9/+^ mouse cells was performed in six-well plates as mentioned above. After transduction, transduced cells were sorted for YFP (for MLL-AF9) or selected with neomycin (for MLL-ENL).

### Dissection and culture of primary mouse prostate tissue

Normal prostate tissue was derived from *Rosa26*-LSL-Cas9 knockin mice on B6J (strain 26175, Jackson Laboratory), and *Pten*^−/−^;*Trp53*^−/−^ prostate tumors were derived from a mouse model of prostate cancer of the same genetic background similar to Feng et al.^57^. Tissues were minced into small pieces and transferred into a gentleMACS C tube (Miltenyi Biotec) for enzymatic digestion using the Multi Tissue Dissociation Kit 1 (130-110-201, Miltenyi Biotec). Digestion was performed in a gentleMACS Dissociator using the program 37C_Multi_A. Following enzymatic dissociation, the cell suspension was pelleted, resuspended and filtered through a 70‐μm cell strainer. Normal and tumoral dissociated prostate cells were cultured as 3D organoids using the protocol and medium composition described by Drost et al.^58^. Cultures were maintained under these conditions for 6–7 days to enrich for epithelial cells, after which cells were maintained as monolayer cultures on plates coated with collagen I (354236, Corning) using the same medium composition.

### Lentiviral vector production and infection

For virus production, 293FT cells were transfected with the lentiviral vector (lentiCRISPR-v2) either as an empty vector or containing NPM1 guide RNA together with the packaging plasmids psPAX2 (Addgene, 12260) and pMD2.G (Addgene, 12259). The viral supernatant was collected 48 and 72 h after transfection and concentrated overnight at 6,000*g* and 4 °C. A total of 1 × 10^6^ cells and viral supernatant were mixed in 2 ml culture medium supplemented with 8 μg ml^−1^ polybrene (Millipore), followed by spinfection (60 min, 900*g*, 32 °C), and were further incubated overnight at 37 °C. The medium was refreshed on the following day, and the transduced cells were cultured further. Pellets for protein were collected 7 and 9 days after transduction.

### Protein extraction and western blot for the knockout experiment

OCI-AML2 cells were transduced with either NPM1 gRNAs or control gRNAs. Cell pellets were collected on day 7 after transduction and lysed with whole-cell lysis buffer (0.2% Nonidet P-40, 50 mM Tris-HCl, pH 8.0, 450 mM NaCl, 1 mM EDTA), 1× protease inhibitor cocktail 1 (Merck), 1× phosphatase inhibitor 2 (Merck), 1× phosphatase inhibitor 3 (Merck) and 1 mM DTT (Epigentek)) and incubated on ice for 10 min. The lysates were then centrifuged at 20,000*g* and 4 °C for 10 min. The supernatant was transferred to a fresh tube and quantified using the Bradford assay (Bio-Rad). Following quantification, the samples were supplemented with 1× LDS sample buffer (Thermo Fisher Scientific) and 1× Sample Reducing Agent (Thermo Fisher Scientific), and they were then incubated at 70 °C for 10 min. Next, 10 μg protein was loaded. Western blotting was performed using SDS–PAGE gels, and samples were blotted onto a PVDF membrane. It was performed using the following antibodies: anti-NPM1 (FC8791), anti-H3 (Abcam, ab1220) as a loading control at a 1:1,000 dilution and goat anti-mouse IgG H&L, HRP conjugated (Abcam, ab205719) at a 1:10,000 dilution.

### Blood counts

For blood counts, 20 μl blood was collected from the tail vein of mice using a capillary pipette containing anticoagulants (EDTA). The EDTA anticoagulated blood samples were used to obtain a complete blood count with a Vet ABC analyzer (Horiba ABX). Samples were counted no longer than 20 min after blood was drawn.

### Real-time PCR

For Fig. 4a, genomic DNA was extracted from murine PB using the DNeasy Blood and Tissue Kit (Qiagen). Genomic DNA (10 ng) was used, and the levels of *Cas9* and *Gapdh* were analyzed on a QuantStudio 5 real-time PCR instrument (Applied Biosystems) using PowerUp SYBR Green Master Mix (Applied Biosciences). The relative quantification of *Cas9* was performed using the comparative cycle threshold (Ct) method against the housekeeping gene *Gapdh*. The primer sequences are listed in Supplementary Table 3.

### PCR

For Extended Data Fig. 6b, genomic DNA was extracted from murine PB 2 weeks after injection using the DNeasy Blood and Tissue Kit (Qiagen). Primers for *Flt3*^ITD^ were used for PCR amplification of genomic DNA (20 ng). The PCR product was analyzed by agarose gel electrophoresis, stained with GelRed. The 1 kb plus DNA Ladder (NEB) was used.

### Antibody staining and flow cytometry analysis of AML cells

For primary murine AML experiments related to Fig. 2b and Extended Data Figs. 2b and 3b, 6–10-week-old C57BL/6 male mice were injected with 10^6^ primary murine AML cells by intravenous injection from the indicated animal AML models, as described above. Upon animal sickness, BM was isolated and lysed in 0.85% NH_4_Cl for 5 min. Primary antibodies, at a concentration of 0.5 μg per reaction, either anti-NPM1 (mAb2) or the IgG2a isotype control (Bio X Cell), were precomplexed with a 1:1,000 dilution of the secondary antibody goat anti-mouse IgG Alexa Fluor 488 (Abcam) for 30 min. For intracellular staining, cells were first permeabilized with 0.1% Triton X-100 (Sigma) for 10 min at room temperature and rinsed with 2% FBS in 1× PBS. For cell surface staining (‘live cell’), cells were processed directly. BM cells were blocked in 2% FBS in 1× PBS for 30 min on ice and then stained with the precomplexed mix of antibodies as stated above. Cells were washed once with 150 µl of 2% FBS in 1× PBS and resuspended in 2% FBS in 1× PBS containing 0.1 µg ml^−1^ DAPI (Sigma). For intracellular staining, cells were fixed with 4% formaldehyde followed by a wash with 2% FBS in 1× PBS. Flow cytometry analysis was performed using a CytoFLEX instrument (Beckman Coulter) and analyzed using FlowJo (version 10, BD).

For primary murine AML experiments related to Fig. 4d, 6–10-week-old C57BL/6 male mice were sublethally irradiated with a whole-body dose of 5.5 Gy and then injected with 10^6^ primary murine AML cells by intravenous injection from the indicated animal AML model, as described above. On day 15 after transplantation, mice were treated i.p. with a single dose of either 5 mg kg^−1^ of the mouse IgG2a isotype control antibody or 5 mg kg^−1^ of anti-NPM1 (mAb2) antibody. On day 18 after transplantation, PB was isolated and lysed in 0.85% NH_4_Cl for 5 min. Flow cytometric analysis of YFP^+^ cells was performed as above.

For Fig. 2c, K562 cells were transduced with the lentiviral cDNA constructs pKLV-TY1-NPM1-PURO or pKLV-TY1-NPM1c-PURO (mutant NPM1) or an empty pKLV-TY1-PURO vector control. Transduced cells were selected with puromycin, and then live cells were stained with either a mouse anti-B23 NPM1 antibody (Merck) or a mouse anti-Ty1 antibody (Diagenode) for 45 min followed by a staining with a 1:1,000 dilution of the secondary antibody goat anti-mouse IgG Alexa Fluor 488 (Abcam) for 45 min. Cells were washed once with 2% FBS in 1× PBS and finally resuspended in 2% FBS in 1× PBS containing 0.1 µg ml^−1^ DAPI. Flow cytometry analysis was performed as above.

For Extended Data Fig. 2g, K562 and OCI-AML3 cells were incubated with human Fc block (BioLegend, 101319). Afterward, they were incubated with WGA conjugated to fluorescein (Vector Laboratories, FL-1021-5) at a concentration of 1:1,000. When colabeled with FC8791 or mAb2, antibodies were precomplexed with anti-mouse IgG Alexa Fluor 647 at a ratio of 2:1 before being incubated with cells. The final concentration of FC8791 and mAb2 was 5 µg ml^−1^, and the secondary antibody was used at 2.5 µg ml^−1^. Cells were washed after binding, subsequently stained with DAPI and then applied to slides using a Cytospin. Images were obtained on a Leica TCS SP8 microscope.

For AML PDX experiments related to Extended Data Fig. 6e,f, 6–10-week-old SCID-CB17 female mice (Charles River, strain 236) were injected with 10^6^ patient-derived AML cells by intravenous injection. BM was isolated and lysed in 0.85% NH_4_Cl for 5 min. Primary antibodies, at a concentration of 0.5 μg per reaction, either anti-NPM1 (mAb2) or the IgG2a isotype control (Bio X Cell), were precomplexed with a 1:1,000 dilution of the secondary antibody goat anti-mouse IgG Alexa Fluor 488 (Abcam) for 30 min. BM cells were blocked in 2% FBS in 1× PBS for 30 min on ice and then stained with the precomplexed mix of antibodies as stated above. Cells were washed once with 150 µl of 2% FBS in 1× PBS and resuspended in 2% FBS in 1× PBS containing 0.1 µg ml^−1^ DAPI. Flow cytometry analysis was performed as above.

For primary murine experiments related to Fig. 5d, freshly isolated BM from male 20-week-old WT and preleukemic *Npm1*^fl−cA/+^ or moribund *Npm1*^fl−cA/+^;*Flt3*^ITD/+^ mice was used. BM cells were exposed to erythrocyte lysis (BD Pharm Lyse, BD Biosciences), followed by magnetic bead selection of Lin^−^ cells using the Lineage Cell Depletion Kit (Miltenyi Biotec) according to the manufacturer’s instructions. Primary antibodies, at a concentration of 0.5 μg per reaction, either anti-NPM1 (mAb2) or the IgG2a isotype control (Bio X Cell), were precomplexed with a 1:1,000 dilution of the secondary antibody goat anti-mouse IgG Alexa Fluor 488 (Abcam) for 30 min. BM cells were blocked in 2% FBS in 1× PBS for 30 min on ice and then stained with the precomplexed mix of antibodies as stated above. Cells were washed once with 150 µl of 2% FBS in 1× PBS and resuspended in 2% FBS in 1× PBS containing 0.1 µg ml^−1^ DAPI. Flow cytometry analysis was performed as above.

For primary murine AML experiments related to Fig. 5a,b, 6–10-week-old C57BL/6 male mice were sublethally irradiated with a whole-body dose of 5.5 Gy and then injected with 10^6^ primary murine AML cells by intravenous injection from the indicated animal AML model, as described above. Upon animal sickness on day 18 after transplantation, BM was isolated and lysed in 0.85% NH_4_Cl for 5 min. BM cells were resuspended in 10% DMSO in FBS and stored at −80 °C for further applications. BM cells were thawed and suspended in PBS supplemented with 2% FBS and stained with biotin anti-mouse Ly-6A/E (SCA1) (BioLegend), biotin anti-mouse CD127 (BioLegend, 135005), biotin anti-mouse CD3 (BioLegend, 100201), biotin anti-mouse TER-119/erythroid cells (BioLegend, 116203), biotin anti-mouse/human CD45R/B220 (BioLegend, 103203), BV605 anti-mouse GR1 (BioLegend, 108439), BV650 anti-mouse CD11b (BioLegend, 101239), PerCP/Cy5.5 anti-mouse CD16/CD32 (BioLegend, 101323), PE anti-mouse CD93 (BioLegend, 136503), APC anti-mouse CD48 (BioLegend, 103411), APC/Fire 750 anti-mouse CD117 (c-Kit) (BioLegend, 135139), Zombie aqua viability dye (BioLegend, 423101) and BV421 streptavidin (BioLegend, 405226). All the antibodies were used at a 1:400 dilution, apart from the viability dye, which was used at a 1:1,000 dilution. The samples were then stained with either anti-NPM1 (mAb2) antibody or the IgG2a isotype control (Bio X Cell, BE0085), precomplexed with anti-mouse IgG Alexa Fluor 594 (Abcam, ab150108) as a secondary antibody. FMO controls were included in the experiments to provide a measure of spillover in each channel. This allows for correct gating in each experimental sample. Flow cytometry analysis was performed using the Cytek Aurora spectral analyzer and analyzed using FlowJo (version 10, BD). Data in this section were plotted using GraphPad Prism (version 9).

### Antibody staining and flow cytometry analysis of solid cancer models

Primary antibodies, at a concentration of 0.5 μg per reaction, either anti-NPM1 (mAb2) or the IgG2a isotype control (Bio X Cell), were precomplexed with a 1:1,000 dilution of the secondary antibody goat anti-mouse IgG Alexa Fluor 488 (Abcam) for 30 min. A total of 5 × 10^4^ cells for each model used in Fig. 6 (Fig. 6a,b,e,h) were blocked in 2% FBS in 1× PBS for 30 min on ice and then stained with the precomplexed mix of antibodies as stated above. Cells were washed once with 150 µl of 2% FBS in 1× PBS and resuspended in 2% FBS in 1× PBS containing 0.1 µg ml^−1^ DAPI (Sigma). Flow cytometry analysis was performed using the CytoFLEX instrument (Beckman Coulter) and analyzed using FlowJo (version 10, BD).

### In vivo treatment of normal, primary murine AML and PDX models

For experiments related to Extended Data Fig. 4d,e, 16–20-week-old C57BL/6 male mice were used, which were housed at Boston Children’s Hospital. All mouse procedures and protocols were approved by the Animal Care and Use Committee of Boston Children’s Hospital and followed all relevant guidelines and regulations. After euthanasia, PB, liver, spleen and BM were harvested.

For experiments related to Extended Data Fig. 5a,b, 6–10-week-old C57BL/6 male mice were given i.p. injections of either anti-NPM1 (mAb2) or the IgG2a isotype control (Bio X Cell, BE0085) antibody at the indicated doses, once per week for a total of 4 weeks (total of four treatments). Weights were recorded, and PB from the tail vein was collected at the indicated time points. Weight measurements of the indicated mouse organs were taken from all treated cohorts at the end of the study, on the 28th day after initiation of the relevant treatments.

For experiments related to Fig. 4d–f and Extended Data Fig. 5c,d, 6–10-week-old C57BL/6 male mice were sublethally irradiated with a whole-body dose of 5.5 Gy. On day 12 after irradiation, mice were given i.p. injections of either anti-NPM1 (mAb2) or the IgG2a isotype control (Bio X Cell, BE0085) antibody at the indicated doses, once per week for a total of 4 weeks (total of four treatments). Weights were recorded, and PB from the tail vein was collected at the indicated time points. Weight measurements of the indicated mouse organs were taken from all treated cohorts at the end of the study, on the 66th day after irradiation.

For primary murine *Npm1*^c^;*Flt3*^ITD/+^/Cas9 AML experiments related to Fig. 4a–c and Extended Data Fig. 6a, 6–10-week-old C57BL/6 male mice were sublethally irradiated with a whole-body dose of 5.5 Gy and then injected with 10^6^ primary murine AML cells by intravenous injection from the indicated animal AML model, as described above. On day 14 after transplantation, mice were given i.p. injections of either anti-NPM1 (mAb2) or the IgG2a isotype control (Bio X Cell, BE0085) antibody at the indicated doses, once per week for a total of 4 weeks (total four of treatments). Weight measurements of leukemic spleens were taken from each animal after humane endpoints were reached.

For primary murine MLL-AF9/*Flt3*^ITD/+^/Cas9 AML experiments related to Fig. 4g,h, 6–10-week-old NSG male mice were injected with 10^6^ primary murine AML cells by intravenous injection from the indicated animal AML model, as described above. On day 14 after transplantation, mice were given i.p. injections of either anti-NPM1 (mAb2) or the IgG2a isotype control (Bio X Cell, BE0085) antibody at the indicated doses, once per week for a total of 3 weeks (total of three treatments). Weight measurements of leukemic spleens were taken from each animal after humane endpoints were reached.

For xenotransplantation AML experiments related to Fig. 4i, 6–10-week-old SCID-CB17 (Charles River, strain 236) female mice were injected with 2 × 10^6^ OCI-AML3 human AML cells by intravenous injection. On day 14 after transplantation, mice were given i.p. injections of either anti-NPM1 (mAb2) or the IgG2a isotype control (Bio X Cell, BE0085) antibody at the indicated doses, once per week for a total of 3 weeks (total of three treatments).

For AML PDX experiments related to Fig. 4j,k, 6–10-week-old SCID-CB17 (Charles River, strain 236) female mice were injected with 10^6^ patient-derived AML cells by intravenous injection. On day 14 after transplantation, mice were given i.p. injections of either anti-NPM1 (mAb2) or the IgG2a isotype control (Bio X Cell, BE0085) antibody at the indicated doses, once per week for a total of 3 weeks (total of three treatments). PB from the tail vein was collected on day 24 after transplantation.

For primary murine MLL-AF9/*Flt3*^ITD/+^/Cas9 AML experiments related to Fig. 5c, 6–10-week-old C57BL/6 male mice were sublethally irradiated with a whole-body dose of 5.5 Gy and then injected with 10^6^ primary murine AML cells from primary recipients. For the primary recipients (related to Fig. 4d,e and Extended Data Fig. 6c,d), 6–10-week-old C57BL/6 male mice were sublethally irradiated with a whole-body dose of 5.5 Gy and then injected with 10^6^ primary murine AML cells by intravenous injection from the indicated animal AML model, as described above. On day 15 after transplantation, mice were treated i.p. with a single dose of either 5 mg kg^−1^ of mouse IgG2a isotype control antibody or 5 mg kg^−1^ of anti-NPM1 (mAb2) antibody. On day 18 after transplantation, BM was isolated and processed accordingly for secondary transplantation as indicated above. Moreover, on day 18 after transplantation, PB from the tail vein as well as weight measurements of spleens, lungs and livers were taken from each animal after humane endpoints were reached.

All mice used in the study were housed in specific pathogen-free conditions in the UBS animal facilities of the University of Cambridge. All cages were on a 12–12-h light–dark cycle (lights on, 07:30) in a temperature-controlled and humidity-controlled room. Room temperature was maintained at 72 ± 2 °F (22.2 ± 1.1 °C), and room humidity was maintained at 30–70%. The animals were culled when leukemia-associated symptoms occurred or humane endpoints were reached. All animal studies were carried out in accordance with the Animals (Scientific Procedures) Act 1986, UK and approved by the Ethics Committee at the University of Cambridge. Randomization and blinding were not applied. All data in this section were plotted using GraphPad Prism (version 9).

### In vivo treatment of mouse solid tumor models

For primary prostate carcinoma experiments related to Fig. 6c,d, 8–10-week-old C57BL/6 male mice were injected with 10^6^ primary murine prostate carcinoma cells by subcutaneous injection (1:1 ratio of Matrigel and cancer cells) from the indicated animal model, as described above. For colorectal carcinoma experiments related to Fig. 6f,g, 8–10-week-old C57BL/6 male mice were injected with 5 × 10^5^ MC38 cells by subcutaneous injection (1:1 ratio of Matrigel and cancer cells). For melanoma experiments related to Fig. 6i, 8–10-week-old C57BL/6 male mice were injected with 5 × 10^5^ B16F10 cells by subcutaneous injection (1:1 ratio of Matrigel and cancer cells). On days 5, 7 and 9 after transplantation, mice were given i.p. injections of either anti-NPM1 (mAb2) or the IgG2a isotype control (Bio X Cell, BE0085) antibody at the indicated doses (total of three treatments). Tumors were dissected when sizes were approaching or reached the humane end point limit (1.2 cm^2^) as per animal license: on day 17 after transplantation for prostate tumors, on day 13 after transplantation for colorectal carcinoma tumors and on day 11 after transplantation for melanoma. All tumor sizes were measured using a digital caliper (Jodsen). All solid tumor models used in the study were housed in specific pathogen-free conditions in the UBS animal facilities of the University of Cambridge,. All cages were on a 12–12-hour light–dark cycle (lights on, 07:30) in a temperature-controlled and humidity-controlled room. Room temperature was maintained at 72 ± 2 °F (22.2 ± 1.1 °C), and room humidity was maintained at 30–70%. The animals were culled when humane endpoints were reached. All animal studies were carried out in accordance with the Animals (Scientific Procedures) Act 1986, UK and approved by the Ethics Committee at the University of Cambridge. Randomization and blinding were not applied. All data in this section were plotted using GraphPad Prism (version 9).

### Cell surface biotinylation, immunoprecipitation and western blotting

K562 cells were cultured as above. Cell surface protein labeling was accomplished using sulfo-NHS-SS-biotin (APExBIO) as described above, after which crude membrane fractions were isolated. To obtain cytosolic and membrane fractions^24^, suspension cells were directly resuspended in membrane isolation buffer (10 mM HEPES (Thermo Fisher Scientific), 250 mM sucrose (Sigma), 1 mM EDTA) at 5 million cells per 1 ml. Adherent cells were collected off the plate by scraping in ice-cold PBS, pelleted and then similarly resuspended at 5 million cells per 1 ml of membrane isolation buffer. Cells were rested on ice for up to 5 min, moved to a glass Dounce homogenizer (Sigma) and then homogenized using 40–80 strokes to obtain a resuspension of approximately 50% released nuclei. Overdouncing can cause nuclear rupture and contamination of the cytosolic fraction. After douncing, unbroken cells and nuclei were pelleted by centrifuging at 4 °C for 10 min at 700*g*. Supernatants (cytosol and membranes) were carefully transferred to a new tube, and pellets were discarded. The supernatants were again centrifuged at 4 °C for 30 min at 10,000*g*. Most (90%) of the supernatant was removed and saved as cytosolic fractions. The remaining supernatant was discarded, as it was near to the membrane pellet. The membrane pellet was gently washed with 500 µl of ice-cold 1× PBS (the pellet was not resuspended here), the tube was centrifuged briefly, and all supernatant was discarded to leave a cleaned membrane pellet. Finally, the membrane pellet was resuspended in 500 µl CLIP lysis buffer (50 mM Tris-HCl, pH 7.5, 200 mM NaCl (Sigma), 1 mM EDTA, 10% glycerol (Thermo Fisher Scientific), 0.1% NP-40, 0.2% Triton X-100, 0.5% *N*-lauroylsarcosine). Both the cytosolic and membrane lysates were stored at −80 °C for later processing. After isolation and solubilization with CLIP lysis buffer, total protein quantification occurred using the BCA assay. For each sample, 5 μg protein was used for input, and 20 μg was used for either anti-NPM1 (FC8791)-coated bead protein A Dynabeads (Thermo Fisher Scientific) or streptavidin-coated bead (MyOne C1 beads, Thermo Fisher Scientific) enrichments. In both cases, 10 μl bead surrey was added to the membrane lysates in 100 μl of CLIP lysis buffer, and binding occurred at 4 °C for 16 h. After binding, the beads were washed three times with high-stringency buffer and then twice with 1× PBS. Proteins were released from the beads by heating at 85 °C for 10 min in 20 µl 1× LDS (Thermo Fisher Scientific) and 1 mM free biotin (Thermo Fisher Scientific). Input and enriched proteins were analyzed by Western blot, staining with anti-NPM1 antibody (Santa Cruz Biotechnology, sc-32256, FC8791) and streptavidin IR800 (LI-COR Biotechnology) and finally scanning on a LI-COR Odyssey CLx scanner. Fractionation as described above was performed without cell surface biotinylation for data in Fig. 1a, and western blots were developing using anti-NPM1 (Santa Cruz Biotechnology, sc-32256, FC8791), anti-NPM1c (Thermo Fisher Scientific, 32-5200), anti-β-actin (Santa Cruz Biotechnology, sc-47778) and anti-RPN1 (Santa Cruz Biotechnology, sc-48367) antibodies.

### mAb2 design and synthesis

VH and VL sequences were grafted to the constant region of the mouse IgG2a heavy chain and the mouse λ light chain, respectively, to generate a new mouse antibody (mAb). These sequences were given to Curia Global. At Curia, the gene synthesis process involves overlapping oligonucleotide synthesis and assembly, followed by cloning into Curia’s proprietary high-expression mammalian vector. Production and mAb quality control were performed by Curia to produce a protein A-purified IgG fraction with low endotoxin (<1 EU per mg IgG) in 137 mM NaCl, 2.7 mM KCl, 10 mM Na_2_HPO_4_, 2 mM KH_2_PO_4_, pH 7.4.

### Live and fixed cell staining (human samples), flow cytometry and data analysis

Cells were cultured as described above and directly counted. Typically, 50,000 cells were used and blocked with Human TruStain FcX (Fc block, BioLegend) or Mouse TruStain FcX (Fc block, BioLegend) in flow cytometry buffer (0.5% BSA (Sigma) in 1× PBS) for at least 15 min on ice; cells were kept on ice from this point forward. For intracellular staining (‘fix–perm’), cells were first fixed with 3.7% formaldehyde for 10 min at 25 °C, rinsed once with 1× PBS and then permeabilized with 0.1% Triton X-100 (Sigma) for 10 min at 25 °C and finally rinsed once with 1× PBS. For cell surface staining (‘live cell’), cells were processed directly after live Fc blocking on ice. Precomplexed antibodies were added to live or fixed cells. To precomplex, primary unconjugated antibodies including mouse isotype (Santa Cruz Biotechnology, sc-2025) and anti-NPM1 (Santa Cruz Biotechnology, sc-32256, FC8791) and anti-Ty1 tag (Diagenode) were bound in solution (precomplexed) to a goat anti-mouse AF647 secondary antibody (Thermo Fisher Scientific, A32728) or a goat anti-mouse AF488 secondary antibody (Thermo Fisher Scientific, A28175) for at least 30 min on ice before use. The molar ratio was 2:1 (primary:secondary). To the blocked cells, precomplexed antibody was added at a final concentration of 1 µg ml^−1^ (primary antibody) and allowed to bind to cells for 60 min on ice. After staining, cells were centrifuged at 4 °C for 3 min at 400*g*, and the supernatant was discarded. All cell centrifugation steps took place using these conditions. Cells were washed once with 150 µl flow cytometry buffer, centrifuged under the same conditions and finally resuspended in flow cytometry buffer containing 0.1 µg ml^−1^ DAPI. Data collection occurred on a BD Biosciences LSRFortessa 3, and a gating strategy was used to isolate live single cells to examine antibody binding using FlowJo software (version 10, BD).

The frozen PB and BM samples obtained from healthy donors as well as patients with AML were processed with care to ensure as little cell lysis and high viability after thawing. Vials were warmed in a 37 °C water bath for 2–3 min and then completely thawed in 5 ml of ice-cold flow cytometry buffer. Cells were pelleted from this initial resuspension at 400*g* for 5 min at 4 °C. The supernatant was discarded, and cells were resuspended in 1 ml of fresh ice-cold flow cytometry buffer and counted. For each sample, 1 million cells per ml were taken for Fc blocking and staining according to the above protocol. Next, we stained the cells using the live protocol with the precomplexed isotype or anti-NPM1 (mAb2) antibodies for 30 min on ice in 100 µl flow cytometry buffer. After that, we centrifuged the cells and discarded the supernatant as before. The cells were then stained again with 200 ng of dye-conjugated cell type-specific antibodies (anti-human CD45 (HI30), 304024; anti-human CD34 (581), 343516; anti-human CD117 (104D2), 313204; anti-human CD33 (WM53), 303416; anti-human CD13 (WM15), 301710; anti-human HLA-DR (L243), 307604; anti-human CD3 (OKT3), 317308; and/or anti-human CD19 (HIB19), 302206; all BioLegend) on ice for 30 min in 100 µl flow cytometry buffer. Finally, the cells were pelleted again, the supernatant was discarded, one 200-µl flow cytometry buffer wash was performed, and the cells were finally resuspended in 200 µl flow cytometry buffer with 0.1 µg ml^−1^ DAPI for flow cytometry analysis. Healthy donor and AML patient samples were obtained with informed consent under (1) UK ethical approval (IRAS reference 340167, previously 149581, REC 07-MRE05-44). Additionally, healthy donor and AML patient samples were collected from patients located at the Dana-Farber Cancer Institute (DFCI) or Brigham and Women’s Hospital (USA). Samples were then processed and banked in the Pasquarello Tissue Bank in accordance with IRB-22-160 at the DFCI; we obtained assistance from the Hematologic Malignancies Data Repository to identify patient samples from the tissue bank that were bona fide AML. The HMDR also provided relevant information regarding disease characteristics, such as cytogenetics, mutations and immunophenotype. Samples were banked in accordance with DFCI Protocol 01-206: tissue and data collection for research studies in patients with hematologic malignancies, BM disorders and normal donors. Sample characteristics were obtained using DFCI IRB Protocol 22-160. This is a DFCI-specific tissue-banking protocol, which is not available publicly for review, but it can be shared upon request.

### Confocal microscopy sample preparation, data acquisition and analysis

For suspension cells, culturing, counting and Fc blocking were carried out as described above. Samples for ‘live cell’ imaging were processed according to the live cell flow cytometry protocol noted above; however, after staining and washing, cells were fixed with 3.7% formaldehyde (37% stock, Sigma) for 30 min at 25 °C. Primary and secondary antibodies noted above were used but sequentially, rather than precomplexed: primary antibody was added at a final concentration of 2.5 µg ml^−1^ for 45 min on ice in flow cytometry buffer. After staining, cells were washed twice with 1× PBS and then stained with a secondary antibody at a final concentration of 2.5 µg ml^−1^. Secondary stains occurred for 30 min on ice and in the dark, after which cells were washed once with 1× PBS. A final fixation for ‘fix–perm’ samples was performed in parallel with the ‘live cell’ samples with 3.7% formaldehyde in 1× PBS for 30 min at 25 °C in the dark. Nuclei were stained with 0.1 µg ml^−1^ DAPI in flow cytometry buffer for 15 min at 25 °C. Suspension cells were applied to glass slides using a Cytospin centrifuge (Fisher Scientific): this was accomplished by centrifugation at 500*g* for 5 min in a Cytospin 1867. Finally, cells were mounted in ProLong Diamond Antifade Mountant (Thermo Fisher Scientific), and a coverglass was sealed over the sample with nail polish. All samples were then imaged on a Leica TCS SP8 STED ONE microscope. For all experiments, at least three regions of interest were acquired using a ×63 oil-immersion objective across one or more *z* slices. Leica’s line-sequential scanning method was used, and images were acquired at 1,024-by-1,024 resolution with a pinhole size of 1 AU. The DAPI channel was acquired with a PMT detector, while all other channels were imaged using hybrid detectors. For Extended Data Fig. 2i, the DAPI channel was imaged using a hybrid detector, and images were analyzed using ImageJ (version 1.54f). Next, using Imaris Microscopy Image Analysis software (Oxford Instruments), single slices of the confocal stack were analyzed.

### Super-resolution imaging and reconstruction

Cells were prepared as described above for live cell imaging. Here, primary conjugated antibodies (AF647) were used directly for cell labeling and included mouse isotype (Santa Cruz Biotechnology, sc-24636-AF647) and anti-NPM1 (Santa Cruz Biotechnology, sc-32256-AF647, FC8791). To avoid cell movement during the SR acquisition, cells were immobilized on a glass-bottom plate precoated with poly-l-lysine (Sigma, P4707) and Cell-Tak (Corning, 354240). An overview of the method and processing can be found in Extended Data Fig. 3a. For single-molecule SR microscopy, we used direct stochastic optical reconstruction microscopy. To perform this, the PBS in which the cells were stored was replaced by a reducing oxygen scavenging buffer to induce blinking of fluorophores as described in the literature^59^. The blinking buffer consisted of 2 μl ml^−1^ catalase (Sigma), 10% (wt/vol) glucose (BD Biosciences), 100 mM Tris-HCl (Thermo Fisher Scientific), 560 μg ml^−1^ glucose oxidase (Sigma) and 20 mM cysteamine (Sigma). First, diffraction-limited images were obtained with low-intensity illumination of few W cm^−2^. Next, the laser power was increased to approximately 3 kW cm^−2^. Image acquisition was started after a short delay to ensure that most fluorophores were shelved into a dark state. The exposure time was 50 ms, and approximately 40,000 frames were recorded.

As the data were obtained with an sCMOS camera, which typically exhibit few pixels with deviating sensitivity (‘hot’ and ‘cold’ pixels), the obtained single-molecule data were corrected for individual pixels with abnormally high or low sensitivity first^60^. In total, 4,000 frames of a raw data stack were averaged. Hot and cold pixels, which are a systematic deviation, persist, in contrast to the random single-molecule signals. Each pixel was compared to its neighbors using 8-connectivity. If a deviation of more than 3% from the median of the neighboring pixel was observed, a correction factor that set the pixel to the median of its neighbors was recorded as previously described^61^. Otherwise, the pixel was not considered to be significantly brighter or darker. This yielded a correction mask, which was applied to all frames of the raw data. Finally, the no-light counts were subtracted from the pixel-corrected data.

For localization of single molecules, the Fiji plugin ThunderSTORM was used^62^. Each camera frame was filtered with a B-spline filter of order 3 and scale 2. Local maxima, corresponding to single-molecule signals, were detected with eight-neighborhood connectivity and a threshold of 1.1 or 1.2 times the standard deviation of the first wavelet level. Detected local maxima were fitted with a 2D Gaussian using least squares, and the position was recorded. Next, to account for single-molecule signals being active in multiple frames, merging of localizations was performed, using a maximum distance of 30 nm and a maximum of 5 off frames with no limit regarding on frames. Cross-correlation-based drift correction (magnification, 5; bin size, 5) was performed, followed by filtering of localizations (sigma of the point spread function between 60 and 270 nm, intensity below 37,800 photons, localization uncertainty smaller than 30 nm). For visualization, the final localizations were reconstructed as 2D histograms with a magnification of 5 (corresponding to a pixel size of 17.7 nm).

For cluster analysis, an automated pipeline was established using the raw list of localizations. First, Ripley’s *H* function was calculated on three areas with a large number of well-separated clusters. The resulting preferential cluster size from the three areas (which was, notably, always very similar) was averaged, multiplied by a correction factor of 0.45 and used as the seed radius for the following DBSCAN analysis. This DBSCAN script yielded all individual clusters, the total number of clusters, the average number of points per cluster and the spatial relation between clusters. The identified final clusters were then analyzed with respect to their spatial relation, size and number of localizations. This unbiased approach follows recommended analysis procedures recently described^63^. Crucially, each dataset was subjected to identical postprocessing and cluster analysis steps with no manual intervention, thus avoiding any biases arising from different parameter settings. Custom scripts used for this analysis can be shared upon request.

### Cell surface proximity labeling of proteins, peptide generation and mass spectrometry data analysis

Samples were prepared similarly to the flow cytometry workflow as described above. However, rather than dye-conjugated secondaries, here an HRP-conjugated secondary (Thermo Fisher Scientific, 31430) was used. The isotype (control) or anti-NPM1 (FC8791) (target) primary unconjugated antibody was precomplexed with an appropriate secondary HRP antibody at 2:1 (primary:secondary) for at least 30 min on ice. Cells were grown as biological triplicate cultures, and typically 2.5 million cells were used per replicate per labeling experiment. Cells were collected from culture, washed of culture medium and resuspended on ice-cold flow cytometry buffer to which the Fc blocker was added for at least 15 min. After blocking, cells were adjusted to 1 million cells per ml of flow cytometry buffer, and then the precomplexed antibodies were added for staining at a final concentration of 2.5 µg ml^−1^. Staining occurred for 60 min at 4 °C on rotation, after which cells were pelleted, supernatants were discarded and cells were washed once with ice-cold 1× PBS. This wash is important to remove excess BSA in the flow cytometry buffer. Next, cells were gently but quickly resuspended in 980 µl of 100 µM biotin-phenol (Iris Biotech) in 1× PBS at 25 °C. To this, 10 µl of 100 mM H_2_O_2_ (Sigma-Aldrich) was quickly added, tubes were capped and inverted, and the reaction was allowed to proceed for 2 min at 25 °C. Precisely after 2 min, the samples were quenched by adding sodium azide and sodium ascorbate at a final concentration of 5 mM and 10 mM, respectively. Samples were inverted, and cells were pelleted at 4 °C. Samples were then washed sequentially once with ice-cold flow cytometry buffer and then twice with ice-cold 1× PBS, after which cell pellets were lysed in 500 µl CLIP lysis buffer, briefly sonicated to solubilize chromatin and frozen at −80 °C for later processing.

Once all the proximity labeling was complete, lysates were thawed in batches to perform the following steps in parallel. Total protein amounts were quantified using the BCA assay, and labeling efficiency and consistency were checked using western blotting. For biotin enrichment, we used the streptavidin western QC to determine the biotin signal across all samples first and then calculated the total µg of lysate that was needed from that sample to generate 5,000,000 units of streptavidin IR800 signal on the LI-COR system. This µg value was then used as the input mass for each of the replicates across all the proximity labeling samples for biotin capture and MS preparation. Samples were normalized to a final volume of 500 µl with CLIP lysis buffer, and, to each sample, 100 µl Pierce NeutrAvidin Agarose (Thermo Fisher Scientific) was added, and samples were incubated at 4 °C for 4 h on rotation. Beads were then washed twice with 1 ml of high-stringency buffer and twice with 1 ml of 4 M NaCl in 100 mM HEPES, all at 37 °C. Salts and detergents were then rinsed from the beads by sequentially washing twice with 1 ml 1× PBS, twice with 1 ml of LC–MS-grade water (Fisher Scientific) and finally with 1 ml of 50 mM ammonium bicarbonate. An on-bead trypsin digestion was then set up by adding 200 µl of 50 mM ammonium bicarbonate and 1 µg MS-grade trypsin to each sample and incubating for 16 h at 37 °C with occasional shaking. After digestion, the samples were acidified by adding formic acid at a final concentration of 0.5%. The solution containing released peptides was moved to a new tube, and the beads were rinsed twice with 300 µl LC–MS-grade water to capture any remaining peptides. All elution and wash samples for a given replicate were combined and reduced to a volume of <200 µl with a SpeedVac. Samples were then desalted using a C18 spin column (Thermo Fisher Scientific): preconditioned with 50% methanol in water and washed twice with 5% acetonitrile, 0.5% formic acid in water, the sample was bound twice to the column, and then the columns were washed four times with 0.5% formic acid in water. Finally, peptides were eluted into Protein LoBind tubes (Eppendorf) with two applications of 40 µl of 70% acetonitrile, 0.5% formic acid in water. Organic solvents were removed with a SpeedVac, and samples were fully dried with a lyophilizer. Resulting peptides were analyzed on the timsTOF Pro as described above.

For all peptides generated, we followed the same procedure for MS analysis and peptide database searching. Specifically, a nanoElute was attached in line to a timsTOF Pro equipped with a CaptiveSpray Source (Bruker). Chromatography was conducted at 40 °C through a 25-cm reversed-phase C18 column (PepSep) at a constant flow rate of 0.5 µl min^−1^. Mobile phase A was 98% water, 2% acetonitrile and 0.1% formic acid (vol/vol/vol), and phase B was acetonitrile with 0.1% formic acid (vol/vol). During a 108-min method, peptides were separated by a three-step linear gradient (5% to 30% B over 90 min, 30% to 35% B over 10 min, 35% to 95% B over 4 min), followed by a 4-min isocratic flush at 95% for 4 min before washing and a return to low organic conditions. Experiments were run as data-dependent acquisitions with ion mobility activated in PASEF mode. MS and MS/MS spectra were collected with *m*/*z* 100 to 1,700, and ions with *z* = +1 were excluded. Raw data files were searched using PEAKS Online Xpro 1.7 (Bioinformatics Solutions). The precursor mass error tolerance and fragment mass error tolerance were set to 20 ppm and 0.03, respectively. The trypsin digest mode was set to semi-specific, and missed cleavages were set to 3. The human Swiss-Prot reviewed (canonical) database version 2020_05 (downloaded from UniProt) and the common Repository of Adventitious Proteins (cRAP version 1.0, downloaded from the Global Proteome Machine Organization) totaling 20,487 entries were used. Carbamidomethylation was selected as a fixed modification. Oxidation (M), deamidation (NQ) and acetylation (N terminus) were selected as variable modifications. Raw data files and searched datasets are available on the Mass Spectrometry Interactive Virtual Environment (MassIVE), a full member of the ProteomeXchange Consortium, under the identifier MSV000092211. The complete searched datasets are also available in Supplementary Information.

To identify enriched proteins from these datasets, we took an approach that compared enrichment in the NPM1 to that of isotype antibodies. Results of the database search were first purged of non-human proteins and keratins as previously described. All proteins with fewer than two unique peptides were also filtered out of the list. Next, Excel was used to calculate the mean spectral count for each of the remaining proteins across triplicates. For each protein, the mean spectral counts associated with each antibody probe were divided by the mean spectral counts of the corresponding isotype, creating an enrichment factor of each protein in the proximity labeling over the isotype. Python (version 3.13) was then used to calculate the ratio of total protein isolated from proximity labeling with protein-targeting antibodies divided by that collected with isotype antibodies. Proteins from each set of proximity labeling data with enrichments either less than two or the previously described ratio were filtered from the dataset. The resultant lists comprise the hits associated with each round of proximity labeling. To perform GO term analysis, all proteins observed across all four fractions from each cell line were concatenated, creating a list of background proteins from each of the four cell lines tested. Lists of hits from the membrane hit pulldowns from each cell line were submitted to DAVID (https://david.ncifcrf.gov/) and run against their respective backgrounds. Next, for each category of GO term (BP, CC and MF), the union of the top four terms across all cell lines and their associated sizes and Benjamini values were plotted for comparison of enrichment across cell lines.

### Anti-NPM1 molecular counting

OCI-AML3 cells were collected, washed with flow cytometry buffer and resuspended at 10^6^ cells per ml. They were then blocked with Human TruStain FcX for 15 min with 5 µl of Fc block per million cells. They were washed again afterward. The cells were then partitioned for live cell and fixed cell staining. Cells planned for fixation were stained with 1 µl of fixable violet LIVE/DEAD stain per 10^6^ cells per ml for 30 min. Afterward, cells were washed with flow cytometry buffer and resuspended in 4% PFA for 10 min at room temperature. They were washed with flow cytometry buffer and then resuspended in 0.1% Triton X for 5 min at room temperature. Again, the cells were washed with flow cytometry buffer. Live cells and fixed cells were then incubated with 500 ng FC8791–Alexa Fluor 647 in 100 µl for 30 min. Cells were washed twice afterward, and live cells were finally resuspended in DAPI. For molecular counting, Quantum Alexa Fluor 647 molecules of equivalent soluble fluorochrome beads were used from Bangs Labs. All samples were analyzed on the BD LSRFortessa. Standard curves were generated and fluorescence quantitation was performed as per Bang Labs’ QuickCal analysis tool.

### Cell surface proximity labeling of RNAs and gel analysis

Samples were prepared here in a similar manner as described above for the proximity labeling of proteins with key differences. The HRP-conjugated secondary antibody was exchanged for protein A–HRP (Cell Signaling) and used at the same molar ratio: two primary antibodies per one protein A–HRP molecule. The biotinylation reagent used here was biotin-aniline (Iris Biotech); it was used at a final concentration of 200 µM in 1× PBS for the labeling reaction, and the labeling reaction was allowed to proceed for 3 min at 25 °C. After pelleting the cells from the quenching reaction, cells were directly lysed, and total RNA was isolated as described before^25^. Briefly, RNAzol RT (Molecular Research Center) was used to lyse cell pellets by placing the samples at 50 °C and shaking for 5 min. To phase separate the RNA, 0.4× volumes of water was added, and samples were vortexed, allowed to stand for 5 min at 25 °C and lastly centrifuged at 12,000*g* at 4 °C for 15 min. The aqueous phase was transferred to clean tubes, and 1.1× volumes of isopropanol was added. The RNA was then purified over a Zymo column (Zymo Research). For all column cleanups, we used the following protocol. First, 350 μl of pure water was added to each column, samples were centrifuged at 10,000*g* for 30 s, and the flowthrough was discarded. Next, precipitated RNA from the RNAzol RT extraction (or binding buffer precipitated RNA, below) was added to the columns, which were centrifuged at 10,000*g* for 10–20 s, and the flowthrough was discarded. This step was repeated until all the precipitated RNA was passed over the column once. Next, the column was washed three times in total: once using 400 μl RNA Prep Buffer (3 M guanidine hydrochloride (GuHCl) in 80% ethanol) and twice with 400 μl 80% ethanol. The first two centrifugation steps were at 10,000*g* for 20 s, and the last one was for 30 s. RNA was then treated with proteinase K (Ambion) on the column. Proteinase K was diluted 1:19 in water and added directly to the column matrix and then allowed to incubate on the column at 37 °C for 45 min. The column top was sealed with either a cap or Parafilm to prevent evaporation. After the digestion, the columns were brought to room temperature for 5 min; lowering the temperature is important before proceeding. Next, eluted RNA was centrifuged out into fresh tubes, and a second elution with water was performed. The mucinase StcE (1.5 μg for every 50 μl of RNA, Sigma-Aldrich) was added to the eluate, and samples were placed at 37 °C for 30 min to digest. The RNA was then cleaned up again using a Zymo column. Here, 2× RNA Binding Buffer (Zymo Research) was added, samples were vortexed for 10 s, and then 2× (samples + buffer) of 100% ethanol was added, and samples were vortexed for 10 s. The final RNA was quantified using a NanoDrop. In vitro RNase or sialidase digestions took place by digesting 50 μg total RNA with one of the following: nothing, 4 μl RNase Cocktail (Thermo Fisher Scientific) or 4 μl of α_2-3,6,8,9_-neuraminidase A (NEB) in 1× NEB Glyco Buffer 1 (NEB) for 60 min at 37 °C. After digestion, RNA was purified using a Zymo column as noted above and was then ready for gel analysis.

To visualize the labeled RNA, it was run on a denaturing agarose gel, transferred to a nitrocellulose membrane and stained with streptavidin^25^. After elution from the column as described above, the RNA was combined with 12 μl of Gel Loading Buffer II (95% formamide (Thermo Fisher Scientific), 18 mM EDTA (Thermo Fisher Scientific), 0.025% SDS) with a final concentration of 1× SYBR Gold (Thermo Fisher Scientific) and denatured at 55 °C for 10 min. It is important to not use Gel Loading Buffer II with dyes. Immediately after this incubation, the RNA was placed on ice for at least 2 min. The samples were then loaded into a 1% agarose, 0.75% formaldehyde, 1.5× MOPS Buffer (Lonza) denaturing gel. Precise and consistent pouring of these gels is critical to ensure a similar thickness of the gel for accurate transfer conditions; we aim for solidified gels approximately 1 cm thick. RNA was electrophoresed in 1× MOPS at 115 V for between 34 and 45 min, depending on the length of the gel. Subsequently, the RNA was visualized with a UV gel imager, and excess gel was cut away; leaving ~0.75 cm of gel around the outer edges of sample lanes will improve transfer accuracy. The RNA was transferred with 3 M NaCl, pH 1 (with HCl) to a nitrocellulose membrane for 90 min at 25 °C. After transfer, the membrane was rinsed with 1× PBS and dried on Whatman paper (GE Healthcare). Dried membranes were rehydrated in Intercept Protein-Free Blocking Buffer, TBS (LI-COR Biosciences) for 30 min at 25 °C. After blocking, the membranes were stained using streptavidin IR800 (LI-COR Biosciences) diluted 1:5,000 in Intercept blocking buffer for 30 min at 25 °C. Excess streptavidin IR800 was washed from the membranes using three washes with 0.1% Tween-20 (Sigma) in 1× PBS for 3 min each at 25 °C. The membranes were then briefly rinsed with PBS to remove the Tween-20 before scanning. Membranes were scanned on a LI-COR Odyssey CLx scanner (LI-COR Biosciences).

### Reporting summary

Further information on research design is available in the Nature Portfolio Reporting Summary linked to this article.

## Online content

Any methods, additional references, Nature Portfolio reporting summaries, source data, extended data, supplementary information, acknowledgements, peer review information; details of author contributions and competing interests; and statements of data and code availability are available at 10.1038/s41587-025-02648-2.

## Supplementary information

Reporting Summary

Supplementary Table 1 Statistics collected from SR reconstructions.

Supplementary Table 2 Cell surface proximity labeling proteomics.

Supplementary Table 3 DNA primer names and sequences.

Supplementary Table 4 The 47 human and mouse solid cancer models profiled.

## Source data

Source Data Fig. 1 Unprocessed western blots and gels.

Source Data Fig. 2 Unprocessed western blots and gels.

Source Data Fig. 5 Flow cytometry gating examples.

Source Data Fig. 6 Flow cytometry gating examples.

Source Data Extended Data Fig. 1 Unprocessed western blots and gels.

Source Data Extended Data Fig. 2 Unprocessed western blots and gels.

## Data Availability

All raw data files and searched datasets related to our proteomic experiments are available on MassIVE, a full member of the ProteomeXchange Consortium, under the identifier MSV000092211. Other requests for materials should be directed to the corresponding authors. Source data are provided with this paper.

## Code availability

No custom code was generated in this work. Adobe Illustrator CC (Adobe) was used for the generation of all the figures shown in this study.
