# Supplementary material for: Treatment of acute myeloid leukemia models by targeting a cell surface RNA-binding protein
Source: Nat Biotechnol. 2025 Apr 23;44(3):430–43. doi: 10.1038/s41587-025-02648-2 (PMC7618518; doi:10.1038/s41587-025-02648-2)
Supplement: Supplementary file 1 — Reporting Summary [file 41587_2025_2648_MOESM1_ESM.pdf]

Corresponding author(s): Ryan A. Flynn
Konstantinos Tzelepis

Last updated by author(s): Mar 19 2025

Reporting Summary

Nature Portfolio wishes to improve the reproducibility of the work that we publish. This form provides structure for consistency and transparency in reporting. For further information on Nature Portfolio policies, see our Editorial Policies and the Editorial Policy Checklist.

Statistics

For all statistical analyses, confirm that the following items are present in the figure legend, table legend, main text, or Methods section.

n/a	Confirmed	
☐	☒	The exact sample size (n) for each experimental group/condition, given as a discrete number and unit of measurement
☐	☒	A statement on whether measurements were taken from distinct samples or whether the same sample was measured repeatedly
☐	☒	The statistical test(s) used AND whether they are one- or two-sided<br>*Only common tests should be described solely by name; describe more complex techniques in the Methods section.*
☒	☐	A description of all covariates tested
☐	☒	A description of any assumptions or corrections, such as tests of normality and adjustment for multiple comparisons
☐	☒	A full description of the statistical parameters including central tendency (e.g. means) or other basic estimates (e.g. regression coefficient) AND variation (e.g. standard deviation) or associated estimates of uncertainty (e.g. confidence intervals)
☐	☒	For null hypothesis testing, the test statistic (e.g. F, t, r) with confidence intervals, effect sizes, degrees of freedom and P value noted<br>*Give P values as exact values whenever suitable.*
☒	☐	For Bayesian analysis, information on the choice of priors and Markov chain Monte Carlo settings
☒	☐	For hierarchical and complex designs, identification of the appropriate level for tests and full reporting of outcomes
☐	☒	Estimates of effect sizes (e.g. Cohen's d, Pearson's r), indicating how they were calculated

Our web collection on statistics for biologists contains articles on many of the points above.

Software and code

Policy information about availability of computer code

Data collection	No software outside the below tools was used.
Data analysis	FlowJo (v10,BD) was used for the analysis of flow cytometry experiments<br>GraphPad Prism (version 9) was used for data representation and statistics<br>Image J 1.54f was used for image analysis<br>Adobe Illustrator CC (Adobe) was used for the generation of the figures<br>Python 3.13<br>Imaris Microscopy Image Analysis Software (Oxford Instruments)<br>PEAKS Online Xpro 1.7 (Bioinformatics Solutions Inc)

For manuscripts utilizing custom algorithms or software that are central to the research but not yet described in published literature, software must be made available to editors and reviewers. We strongly encourage code deposition in a community repository (e.g. GitHub). See the Nature Portfolio guidelines for submitting code & software for further information.

Data

Policy information about availability of data

All manuscripts must include a data availability statement. This statement should provide the following information, where applicable:

- Accession codes, unique identifiers, or web links for publicly available datasets
- A description of any restrictions on data availability
- For clinical datasets or third party data, please ensure that the statement adheres to our policy

Raw data files and searched datasets are available on the Mass Spectrometry Interactive Virtual Environment (MassIVE), a full member of the ProteomeXchange consortium under the identifier: MSV000092211

Research involving human participants, their data, or biological material

Policy information about studies with human participants or human data. See also policy information about sex, gender (identity/presentation), and sexual orientation and race, ethnicity and racism.

Reporting on sex and gender	We used sex unbiased panel of healthy donors and AML patients for bone marrow (BM) and peripheral blood (PB).
Reporting on race, ethnicity, or other socially relevant groupings	We used race unbiased panel of healthy and AML donors for BM and PB.
Population characteristics	All patient samples used were pathologically confirmed to have AML, either de novo or secondary. Most samples were acquired agnostic to sex, gender, immunophenotype or genotype. A portion of samples were selected based on having NPM1 mutations. For the AML patient samples belonging to set A-C illustrated in this manuscript, we had accessibility in the gender and age of patients and we used 26 males and 15 females and age was between 16-89. Age range for all the healthy donor samples were between 0-89.
Recruitment	*Describe how participants were recruited. Outline any potential self-selection bias or other biases that may be present and how these are likely to impact results.*
Ethics oversight	Healthy donor samples and AML patient samples were obtained with informed consent under:<br>1) UK ethical approval (REC 07-MRE05-44). IRAS ref: 340167 (previously 149581).<br>2) Samples were collected from patients located at the Dana Farber Cancer Institute or Brigham and Women's Hospital (USA). Samples are then processed and banked in the Pasquerello Tissue Bank in accordance with IRB-22-160 at DFCI, we obtained assistance from Hematologic Malignancies Data Repository to identify patient samples from the tissue bank that were bona fide AML. HMDR also provided relevant information regarding disease characteristics, such as cytogenetics, mutations and immunophenotype.<br>Samples were banked in accordance with the DFCI Protocol 01-206: tissue and data collection for research studies in patients with hematologic malignancies, bone marrow disorders and normal donors. Sample characteristics were obtained using DFCI IRB protocol 22-160. This is a DFCI specific tissue banking protocol, which is not available publicly for review but it can be shared upon request.

Note that full information on the approval of the study protocol must also be provided in the manuscript.

Field-specific reporting

Please select the one below that is the best fit for your research. If you are not sure, read the appropriate sections before making your selection.

☒ Life sciences ☐ Behavioural & social sciences ☐ Ecological, evolutionary & environmental sciences

For a reference copy of the document with all sections, see nature.com/documents/nr-reporting-summary-flat.pdf

Life sciences study design

All studies must disclose on these points even when the disclosure is negative.

Sample size	No statistical tests were used to determine sample size; they were determined based on the standard in the field and on the numbers required to achieve statistical significance. Replication between biological samples was robust. For each figure the sample size as, independent biological replicates are specified in the legend. For animal experiments we used 5 animals per condition in each experiment to achieve significance apart from one experiment were we used 3 animals (biological replicates). For flow cytometry experiments we used in depended cancer models per tissue as biological replicates in addition to the replicates used per model (3 replicates).The above strategies are standard in the field and are sufficient to achieve statistical significance.
Data exclusions	None
Replication	Experiments in this study were performed 3 independent times, when applicable, and all attempts at replication were successful. Experiments

Replication	using human primary samples could not be repeated due to the availability of the material, each human primary sample is treated as an individual biological replicate.
Randomization	No randomization has taken place in this study apart from the animal experiments, when multiple groups were littermates of the same sex and were randomly assigned to experimental groups. All human samples (healthy and AML) were obtained from an unbiased panel agnostic to sex or gender. For the rest of the experiments we used well-established cancer cell lines, no randomisation is needed as we only have one group of cancer cell lines.
Blinding	All data collection and quantification relied on well established and widely used software (flow cytometry)

Reporting for specific materials, systems and methods

We require information from authors about some types of materials, experimental systems and methods used in many studies. Here, indicate whether each material, system or method listed is relevant to your study. If you are not sure if a list item applies to your research, read the appropriate section before selecting a response.

Materials & experimental systems

n/a	Involved in the study
☐	☒ Antibodies
☐	☒ Eukaryotic cell lines
☒	☐ Palaeontology and archaeology
☐	☒ Animals and other organisms
☒	☐ Clinical data
☒	☐ Dual use research of concern
☒	☐ Plants

Methods

n/a	Involved in the study
☒	☐ ChIP-seq
☐	☒ Flow cytometry
☒	☐ MRI-based neuroimaging

Antibodies

Antibodies used

Antibodies used are in the method section, including information about provider, catalogue number and fluorophore
anti-B23 NPM1 antibody Merck B0556
anti-mouse CD3 BioLegend 100201
anti-mouse IgG Alexa Fluor 594 Abcam ab150108
anti-NPM1 AF647, FC8791 Santa Cruz Biotechnology sc-32256 AF647 0.5
anti-NPM1, FC8791 Santa Cruz Biotechnology sc-32256
anti-NPM1c ThermoFisher Scientific 32-5200
anti-RPN1 Santa Cruz Biotechnology sc-48367
anti-TY1 antibody Diagenode C15200054 0.5 ug per reaction
APC anti-mouse CD48 BioLegend 103411
APC/Fire 750 anti-mouse CD117, c-kit BioLegend 135139
biotin anti-mouse CD3 BioLegend 100243
Biotin anti-mouse Ly-6A/E (Sca-1) Biolegend 108103
biotin anti-mouse TER-119/Erythroid cells BioLegend 116203
biotin anti-mouse/human CD45R/B220 BioLegend 103203
BV605 anti-mouse Gr-1 BioLegend 108439
BV650 anti-mouse CD11b BioLegend 101239
CD127 BioLegend 135005
anti-human CD45 (HI30), AF700 BioLegend 304024
anti-human CD34 (581), PE-Cy7 BioLegend 343516
anti-human CD117 (104D2), PE BioLegend 313204
anti-human CD33 (WM53), BV421 BioLegend 303416
anti-human CD13 (WM15), APC-Cy7 BioLegend 301710
anti-human HLA-DR (L243), FITC BioLegend 307604
anti-human CD3 (OKT3), PE BioLegend 317308
anti-human CD19 (HIB19), FITC BioLegend 302206
Goat anti Mouse IgG (H+L) Secondary Antibody, HRP Thermo Fischer Scientific 31430
Goat anti-Mouse AF488 secondary antibody ThermoFisher Scientific A28175
Goat anti-Mouse AF647 secondary antibody ThermoFisher Scientific A32728
Goat Anti-Mouse IgG, Alexa Fluor 488 Abcam ab150113
IgG2a-isotype control BioXCell BE0085 5ug per 100 ul reaction
Mouse IgG1,k Isotype AF647 Santa Cruz Biotechnology sc-24636 AF647 5 ug per 100 ul reaction
Normal mouse IgG Santa Cruz Biotechnology sc-2025 5 ug per 100 ul reaction
PE anti-mouse CD93 BioLegend 136503
PerCP/Cy5.5 anti-mouse CD16/32 BioLegend 101323
anti-H3 abcam ab1220
Goat anti-mouse IgG H&L HRP, Abcam, ab205719

Validation

All antibodies were validated by and purchased from commercial vendors and were used according to the manufacturer's instructions. The newly developed mAb2 was validated as shown in figure S2 C, D, E, F, J.

anti-B23 NPM1 antibody Merck B0556 : validate by supplier with the following notes- species reactivity rat, kangaroo rat, canine, bovine, human, mouse, monkey, hamster, application immunocytochemistry, immunoprecipitation, ELISA, western blot
anti-mouse CD3 BioLegend 100201: validated by supplier with the following notes- species reactivity mouse and application FC-quality tested, IHC-F- verified
anti-NPM1 FC8791 Santa Cruz Biotechnology sc-32256 validated by the manufacturer with the notes- FC-8791 is recommended for detection of B23 of mouse, rat and human by Western Blotting, Immunofluorescence
anti-NPM1c ThermoFisher Scientific 32-5200 validated by the manufacturer with the following notes- species reactivity human, mouse rat,and applications western blot, IHC, ICC/IF, ELISA, IP, RIP
anti-RPN1 Santa Cruz Biotechnology sc-48367 validated by supplier with the following notes- species reactivity mouse rat and human, applications WB, IP, IF, ELISA
anti-TY1 antibody Diagenode C15200054 validated by supplier with the following notes: monoclonal antibody raided in mouse against the Ty1 tag applications ChIP and western blotting.
APC anti-mouse CD48 BioLegend 103411: verified by supplier with the following notes: verified reactivity mouse and applications FC-quality tested
APC/Fire 750 anti-mouse CD117, c-kit BioLegend 135139 verified by supplier with the following notes: verified reactivity mouse and applications FC- quality tested
biotin anti-mouse CD3 BioLegend 100243 verified by supplier with the following notes: verified reactivity mouse and applications FC-quality tested
Biotin anti-mouse Ly-6A/E (Sca-1) Biolegend 108103 verified by supplier with the following notes: verified reactivity mouse and applications FC- quality tested
biotin anti-mouse TER-119/Erythroid cells BioLegend 116203 verified by supplier with the following notes: verified reactivity mouse and applications FC- quality tested
biotin anti-mouse/human CD45R/B220 BioLegend 103203 verified by supplier with the following notes: verified reactivity mouse and human and applications FC- quality tested IHC-F-verified
BV605 anti-mouse Gr-1 BioLegend 108439 verified by supplier with the following notes: verified reactivity mouse and applications FC- quality tested
BV650 anti-mouse CD11b BioLegend 101239 verified by supplier with the following notes: verified reactivity mouse, human, cynomolgus, rhesus and applications FC-quality tested
CD127 BioLegend 135005 verified by supplier with the following notes: verified reactivity mouse and applications FC- quality tested
anti-human CD45 (HI30), AF700 BioLegend 304024 verified by supplier with the following notes: verified reactivity human and applications FC- quality tested
anti-human CD34 (581), PE-Cy7 BioLegend 343516 verified by supplier with the following notes: verified reactivity human and applications FC- quality tested
anti-human CD117 (104D2), PE BioLegend 313204 verified by supplier with the following notes: verified reactivity human and applications FC- quality tested SB-reported in the literature not verified in mouse
anti-human CD33 (WM53), BV421 BioLegend 303416 verified by supplier with the following notes: verified reactivity human and applications FC- quality tested
anti-human CD13 (WM15), APC-Cy7 BioLegend 301710 verified by supplier with the following notes: verified reactivity human and applications FC- quality tested
anti-human HLA-DR (L243), FITC BioLegend 307604 verified by supplier with the following notes: verified reactivity human, cynomolgus and Rhesus, and applications FC- quality tested
anti-human CD3 (OKT3), PE BioLegend 317308 verified by supplier with the following notes: verified reactivity human and applications FC- quality tested
anti-human CD19 (HIB19), FITC BioLegend 302206 verified by supplier with the following notes: verified reactivity human and applications FC- quality tested
IgG2a-isotype control BioXCell BE0085 Verified by supplier for use as a non-reactivity isotype-matched control for mouse IgG2a antibodies in most in vitro and in vivo applications
Mouse IgG1,k Isotype Santa Cruz Biotechnology sc-24636 Verified by the manufacturer with the following notesnormal mouse IgG1 Alexa Fluor® 647 is recommended for use as an isotype control immunoglobulin in place of a target specific primary antibody of the same isotype (mouse IgG1) by flow cytometry.
Normal mouse IgG Santa Cruz Biotechnology sc-2025 verified by supplier, is an uncojjuated conjugated isotype control immunoglonulin from mouse, recommended for use as an isotype control immunoglobulin in place of a target specific primary antibody of the same isotype (mouse IgG) by WB IP and IHC.
PE anti-mouse CD93 BioLegend 136503 verified by supplier with the following notes: verified reactivity mouse and applications FC-quality tested
PerCP/Cy5.5 anti-mouse CD16/32 BioLegend 101323 verified by supplier with the following notes: verified reactivity mouse and applications FC- quality tested
anti-H3: validated by manufacturer for ICC/IF WB ChIP and IHF suitable for human samples

Eukaryotic cell lines

Policy information about cell lines and Sex and Gender in Research

Cell line source(s)

OCI-AML3 Sanger Intitute Cancer Cell Collection
K562 ATCC
OCI-AML2 Sanger Intitute Cancer Cell Collection
Nalm6 ATCC
MOLM-13 Sanger Intitute Cancer Cell Collection
Jurkat ATCC
SupT1 ATCC
Jeko-1 ATCC
Kasumi-1 Sanger Intitute Cancer Cell Collection
HEK293T ATCC
A549 Sanger Intitute Cancer Cell Collection
NCI-H520 Sanger Intitute Cancer Cell Collection

Calu-1 Sanger Intitute Cancer Cell Collection
HCT-116 Sanger Intitute Cancer Cell Collection
MC-38 Sanger Intitute Cancer Cell Collection
A-673 Sanger Intitute Cancer Cell Collection
MHH-ES-1 Sanger Intitute Cancer Cell Collection
U2OS Sanger Intitute Cancer Cell Collection
HOS Sanger Intitute Cancer Cell Collection
MG-G3 Sanger Intitute Cancer Cell Collection
SaOS Sanger Intitute Cancer Cell Collection
KYSE-30 Sanger Intitute Cancer Cell Collection
OE-21 ATCC
KYSE-140 Sanger Intitute Cancer Cell Collection
OE33 ATCC
FADU ATCC
DETROIT ATCC
UT-SSC-42B Sanger Intitute Cancer Cell Collection
SiMa Sanger Intitute Cancer Cell Collection
SH-SY5Y Sanger Intitute Cancer Cell Collection
LA-N-5 Sanger Intitute Cancer Cell Collection
KNS-42 ATCC
H4 ATCC
U251 ATCC
PFSK1 Sanger Intitute Cancer Cell Collection
MDA-MB-231 Sanger Intitute Cancer Cell Collection
HT1080 Sanger Intitute Cancer Cell Collection
PC3 Sanger Intitute Cancer Cell Collection
22Rv1 Sanger Intitute Cancer Cell Collection
This cell line was derived as published in https://doi.org/10.1073/pnas.2110344118 (reference citation 69 in main text)
ASPC3 Sanger Intitute Cancer Cell Collection
BXPC3 Sanger Intitute Cancer Cell Collection
PANC1 Sanger Intitute Cancer Cell Collection
SU86.86 Sanger Intitute Cancer Cell Collection
YAPC Sanger Intitute Cancer Cell Collection
HPAF-II Sanger Intitute Cancer Cell Collection
PDAC1 Kindly provided by Roland Rad as published in 10.1038/nature25459. Epub 2018 Jan 24. (reference citation 68 in main text)
PDAC2 Kindly provided by Roland Rad as published in 10.1038/nature25459. Epub 2018 Jan 24. (reference citation 68 in main text)
PDAC3 Kindly provided by Roland Rad as published in 10.1038/nature25459. Epub 2018 Jan 24. (reference citation 68 in main text)
PDAC4 Kindly provided by Roland Rad as published in 10.1038/nature25459. Epub 2018 Jan 24. (reference citation 68 in main text)
PDAC5 Kindly provided by Roland Rad as published in 10.1038/nature25459. Epub 2018 Jan 24. (reference citation 68 in main text)
PDAC6 Kindly provided by Roland Rad as published in 10.1038/nature25459. Epub 2018 Jan 24. (reference citation 68 in main text)
PDAC7 Kindly provided by Roland Rad as published in 10.1038/nature25459. Epub 2018 Jan 24. (reference citation 68 in main text)
PDAC8 Kindly provided by Roland Rad as published in 10.1038/nature25459. Epub 2018 Jan 24. (reference citation 68 in main text)
PDAC9 Kindly provided by Roland Rad as published in 10.1038/nature25459. Epub 2018 Jan 24. (reference citation 68 in main text)
PDAC10 Kindly provided by Roland Rad as published in 10.1038/nature25459. Epub 2018 Jan 24. (reference citation 68 in main text)
B16F10 Sanger Intitute Cancer Cell Collection

Authentication

Cell lines have been checked for morphology as instructed by ATCCA.

Mycoplasma contamination

All lines were routinely tested for mycoplasma by PCR and they were negative.

Commonly misidentified lines (See ICLAC register)

No commonly misidentified lines were used in this study.

Animals and other research organisms

Policy information about studies involving animals; ARRIVE guidelines recommended for reporting animal research, and Sex and Gender in Research

Laboratory animals

Flt3ITD/+ mice Gary Gilliland (female 6-10 weeks)
Npm1flox-cA/+, strain George Vassiliou lab, University of Cambridge (female 6-10 weeks)
Rosa26 Cas9/+, George Vassiliou Lab, University of Cambridge (female 6-10 weeks)
C57BL/6 mice, Boston Children's Hospital
C57BL/6 mice, University of Cambridge (6-10weeks)

	NSG, Strain ID: 614, Charles River (6-10 weeks)<br>CB17-SCID, Strain ID: 236, Charles River (6-10 weeks)
Wild animals	No wild animals were used in this study
Reporting on sex	Both male and female mice were used in this study. For<br>Figure 5 A,B,C male C57BL/6 mice were used<br>For figure 5 D E F C57BL/6 female mice were used<br>For figure 5 G H NSG, Strain ID: 614 Charles River female mice were used<br>For figure 5I CB17-SCID, Strain ID female mice were used<br>For figure 5 J K CB17-SCID, Strain ID female mice were used<br>For figure 6 H, I NSG, Strain ID male were used<br>For figure 7 C57BL/6 male mice were used
Field-collected samples	No field collected samples were used in this study
Ethics oversight	All animal studies were carried out in accordance with the Animals (ScientificProcedures) Act 1986, UK and approved by the Ethics Committee at the University of Cambridge.

Note that full information on the approval of the study protocol must also be provided in the manuscript.

Plants

Seed stocks	N/A
Novel plant genotypes	N/A
Authentication	N/A

Flow Cytometry

Plots

Confirm that:

☒ The axis labels state the marker and fluorochrome used (e.g. CD4-FITC).

☒ The axis scales are clearly visible. Include numbers along axes only for bottom left plot of group (a 'group' is an analysis of identical markers).

☒ All plots are contour plots with outliers or pseudocolor plots.

☒ A numerical value for number of cells or percentage (with statistics) is provided.

Methodology

Sample preparation	For primary murine AML experiments animlas were collected upon sickness and BM was isolated and lysed in 0.85% NH4Cl for 5 minutes before proceeding with the staining. For human and murine cell lines the cells were blocked in 2% FBS prior to staining. Primary antibodies, in a concentration of 0.5 µg per reaction, either anti-NPM1 (mAb2) or IgG2a-isotype control (BioXCell) were pre-complexed with 1:1000 dilution of the secondary antibody Goat Anti-Mouse IgG, Alexa Fluor 488 (Abcam) for 30 minutes. For the intracellular staining cells were first permeabilized with 0.1% Triton X100 (Sigma) for 10 minutes at room temperature and rinsed with 2% FBS in 1x PBS. For cell surface staining ("Live cell") cells were processed directly. Bone marrow cells were blocked in 2% FBS in 1x PBS for 30 min on ice and then stained with the pre-complexed mix of antibodies as stated above. Cells were washed once with 150 µL of 2% FBS in 1x PBS, resuspended in 2% FBS in 1x PBS containing 0.1 µg/mL 4',6-diamidino-2-phenylindole (DAPI, Sigma). For intracellular staining cells were fixed with 4% formaldehyde followed by a wash with 2% FBS in 1x PBS.<br>For primary murine AML experiments related to figure 6G, H, BM was collected upon animal sickness and lysed in 0.85% NH4CL for 5 minutes. BM cells were resuspended in 10% DMSO in FBS and stored in -80? for further applications. BM cells were thawed suspended in PBS supplemented with 2% FBS and stained with Biotin anti-mouse Ly-6A/E (Sca-1) (Biolegend), biotin anti-mouse CD127 (Biolegend, cat. no. 135005), biotin anti-mouse CD3 (Biolegend, cat. no. 100201), biotin anti-mouse TER-119/Erythroid cells (Biolegend, cat. no. 116203), biotin anti-mouse/human CD45R/B220 (Biolegend, cat. no. 103203), BV605 anti-mouse Gr-1 (Biolegend, cat. no. 108439), BV650 anti-mouse CD11b (Biolegend, cat. no. 101239), PerCP/Cy5.5 anti-mouse CD16/32 (Biolegend, cat. no. 101323), PE anti-mouse CD93 (Biolegend, cat. no. 136503), APC anti-mouse CD48 (Biolegend, cat. no. 103411), APC/Fire 750 anti-mouse CD117 (c-kit) (Biolegend, cat. no. 135139), Zombie aqua viability dye (Biolegend, cat. no. 423101), and BV421 streptavidin (Biolegend, cat. no. 405226). The samples were then stained for either anti-NPM1 (mAb2) antibody or IgG2a-isotype control (BioXCell, BE0085), pre-complexed with an anti-mouse IgG Alexa Fluor 594 (Abcam, cat.no. ab150108) as a secondary antibody. FMO controls were included in the experiments to provide a

measure of spillover in each channel. This allows for correct gating in each experimental sample.
For live and fixed cell staining of human samples: . Typically, 50,000 cells were used and blocked with Human TruStain FcX (Fc block, BioLegend) or Mouse TruStain FcX (Fc block, BioLegend) in FACS buffer (0.5% BSA (Sigma) in 1x PBS) for at least 15 minutes on ice, cells were kept on ice from this point forward. For intracellular staining ("Fix/Perm") cells were first fixed with 3.7% formaldehyde for 10 minutes at 25?, rinsed once with 1x PBS, then permeabilized with 0.1% Triton X100 (Sigma) for 10 minutes at 25? and finally rinsed once with 1x PBS. For cell surface staining ("Live cell") cells were processed directly after the live Fc blocking on ice. To live or fixed cells, precomplexed antibodies were added. To precomplex, primary unconjugated antibodies including Mouse isotype (Santa Cruz Biotechnology, sc-2025), and anti-NPM1 (Santa Cruz Biotechnology, sc-32256; FC8791), and anti-Ty1 tag (Diagenode) were bound in solution (precomplexed) to a Goat anti-Mouse AF647 secondary antibody (ThermoFisher Scientific, A32728) or a Goat anti-Mouse AF488 secondary antibody (ThermoFisher Scientific, A28175) for at least 30 minutes on ice before using. The molar ratio was 2:1, primary:secondary. To the blocked cells, precomplexed antibody was added to a final concentration of 1 µg/mL (primary antibody) and allowed to bind to cells for 60 minutes on ice. For the frozen peripheral blood (PB) and bone marrow (BM) samples obtained from healthy donors as well as AML patients were processed with care to ensure as little cell lysis and high viability after thawing. Vials were warmed in a 37? water bath for 2-3 minutes and then completely thawed in 5 mL of ice cold FACS buffer. Cells were pelleted from this initial resuspension at 400 x g for 5 minutes at 4?. The supernatant was discarded, cells were resuspended in 1 mL of fresh ice cold FACS buffer and counted. For each sample 1M cells per mL were taken for Fc blocking and staining as per the above protocol. Next we stained the cells using the live protocol with the precomplexed isotype or anti-NPM1 (mAb2) antibodies for 30 minutes on ice in 100 µL FACS buffer. After which we spun the cells down and discarded the supernatant as before. The cells were then stained again with dye-conjugated cell-type specific antibodies (anti-human CD45 (HI30), 304024; anti-human CD34 (581), 343516; anti-human CD117 (104D2), 313204; anti-human CD33 (WM53), 303416; anti-human CD13 (WM15), 301710; anti-human HLA-DR (L243), 307604; anti-human CD3 (OKT3), 317308; and/or anti-human CD19 (HIB19), 302206; all BioLegend) on ice for 30 minutes in 100 µL FACS buffer. Finally the cells were pelleted again, supernatant discarded, once 200 µL FACS buffer wash was performed, and the cells were finally resuspended in 200 uL of FACS buffer with 0.1 µg/mL DAPI for FACS analysis.

Instrument

BD Biosciences LSRFortessa 3
Cytek Aurora Spectra Analyser
Cytoflex (Beckman Coulter)

Software

FlowJo (v10,BD)

Cell population abundance

Abundance of population of interest ranged from 1 to >95% depending on the state of the cells.

Gating strategy

For experiments in figures 2A 2B,2C S2B,S3B, S5D, S5E, 7A, 7B, 7E, 7H the gating strategy was the following:
1.FSC-A/SS-A showing all events gate is placed on cell population to exclude debris and dead cells
2. Single cell gating gating on singlets to exclude doublets
3. Live cell gating (with a viability marker) gate is placed on live cells (negative population) to exclude dead cells
4. Sub-population gating for csNPM1 gate was placed based on the staining of the IgG control.

For figures involving human primary samples healthy and diseased gating strategy is included in the figure.
For figures involving mouse primary samples the gating strategy is provided in the supplementary information.
FMOs and Single stains were used in all the panels in order to ensure correct positive and negative gating

☒ Tick this box to confirm that a figure exemplifying the gating strategy is provided in the Supplementary Information.
