# Supplementary figures and images for: Treatment of acute myeloid leukemia models by targeting a cell surface RNA-binding protein
Source: Nat Biotechnol. 2025 Apr 23;44(3):430–43. doi: 10.1038/s41587-025-02648-2 (PMC7618518; doi:10.1038/s41587-025-02648-2)

Figure 1

C

sulfo-NHS-SS-biotinylated K562s

Mem lysate  
NPM1 IP

Mem lysate  
NPM1 IP

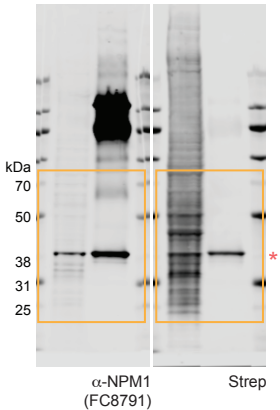

Supplement: Supplementary file 6 — Unprocessed western blots and gels. [file 41587_2025_2648_MOESM6_ESM.pdf]

Figure 2

F

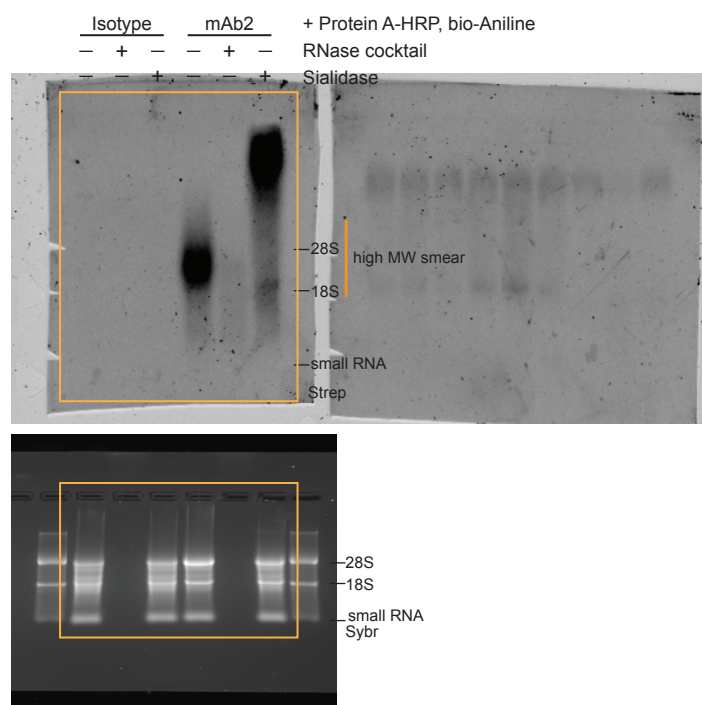

Supplement: Supplementary file 7 — Unprocessed western blots and gels. [file 41587_2025_2648_MOESM7_ESM.pdf]

A / B

gating strategy

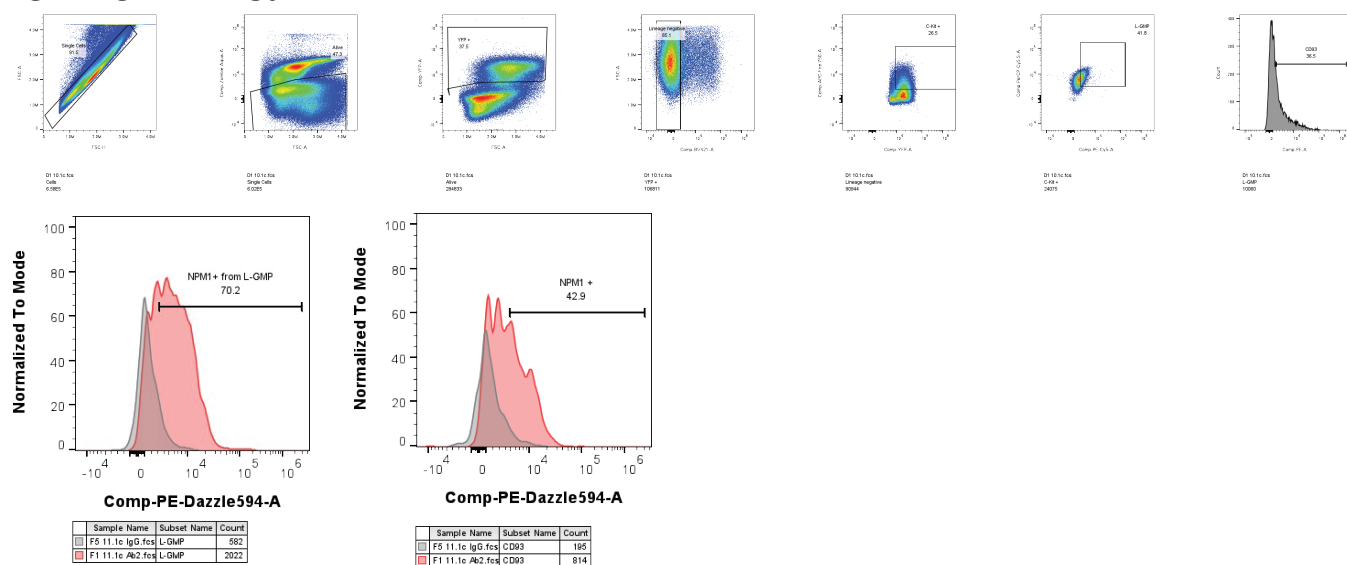

D

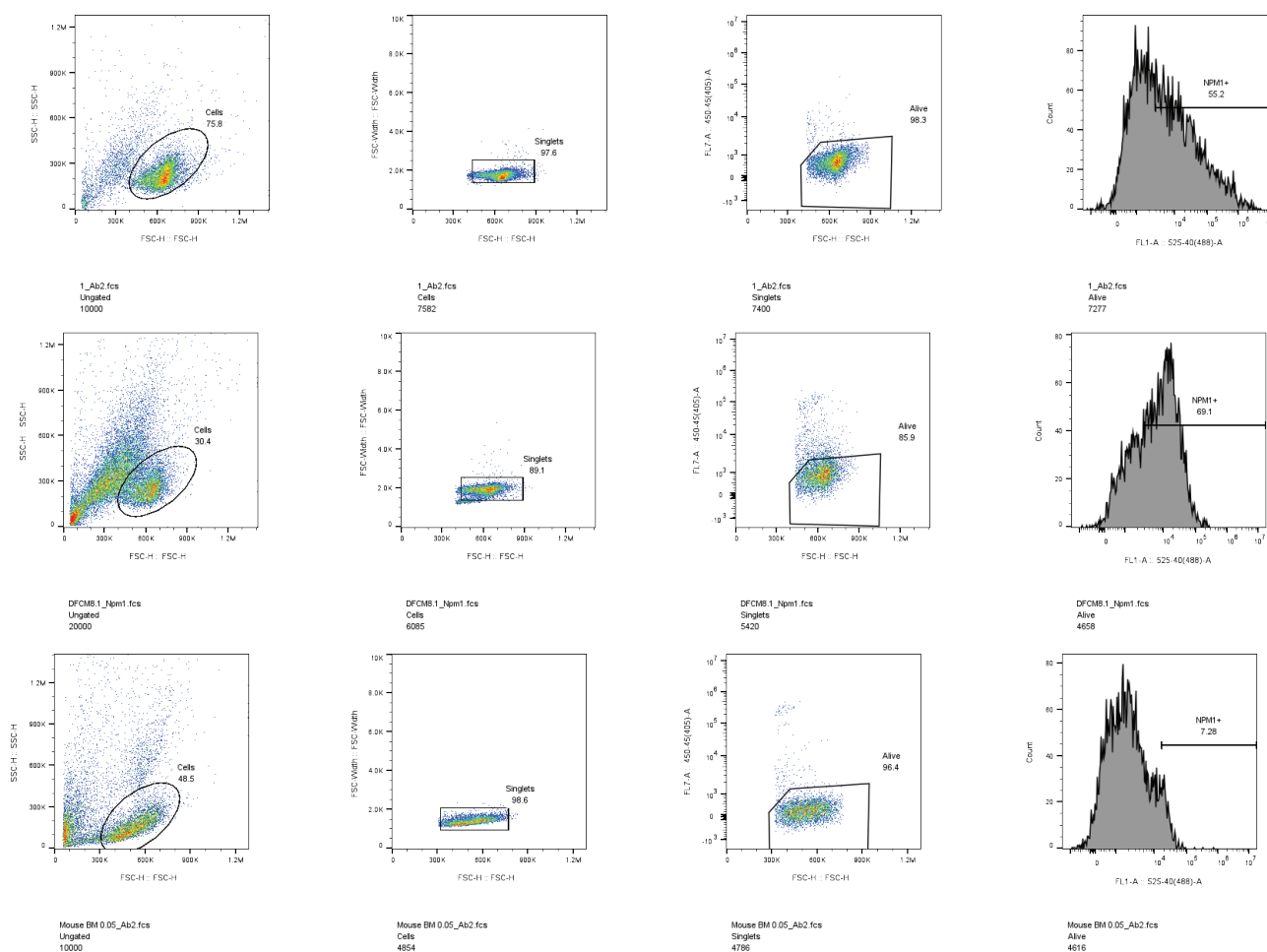

Supplement: Supplementary file 8 — Flow cytometry gating examples. [file 41587_2025_2648_MOESM8_ESM.pdf]

Figure 6

B

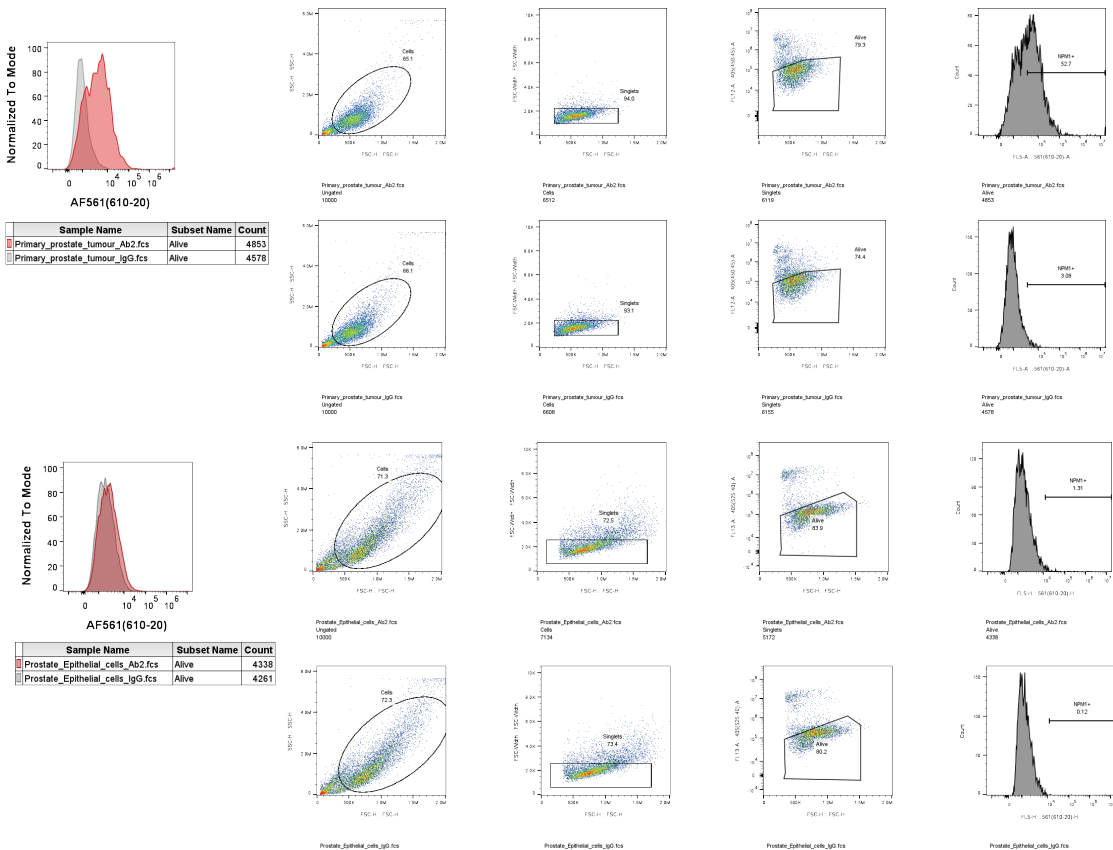

E

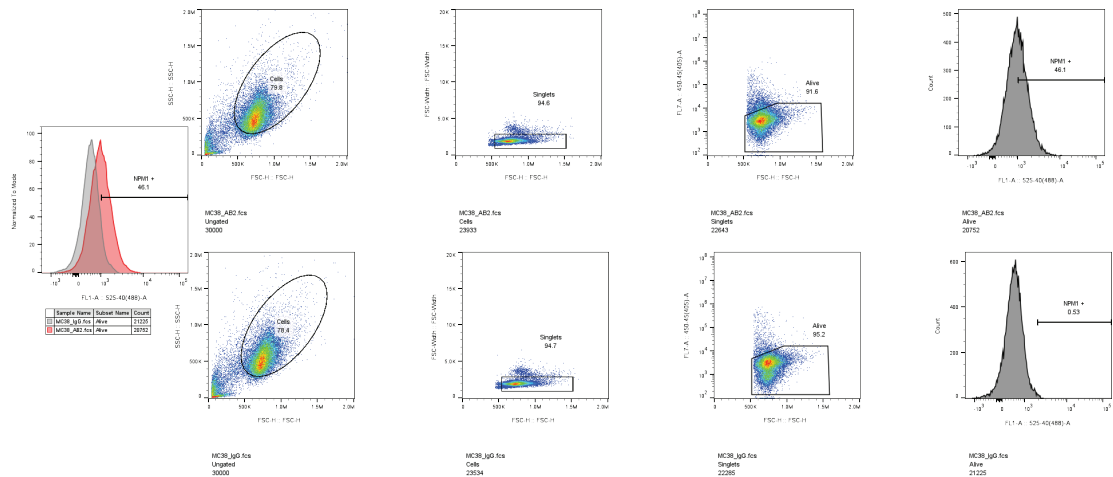

H

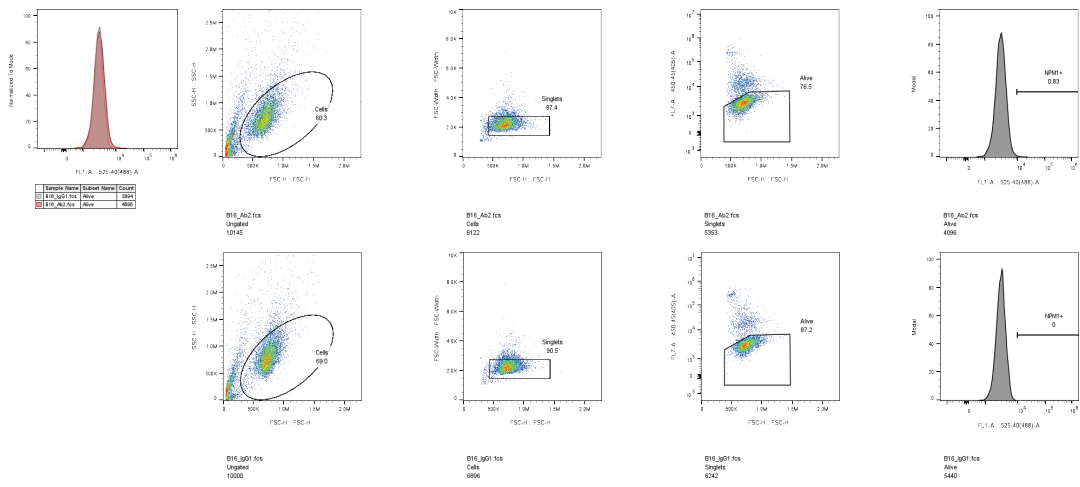

Supplement: Supplementary file 9 — Flow cytometry gating examples. [file 41587_2025_2648_MOESM9_ESM.pdf]

B

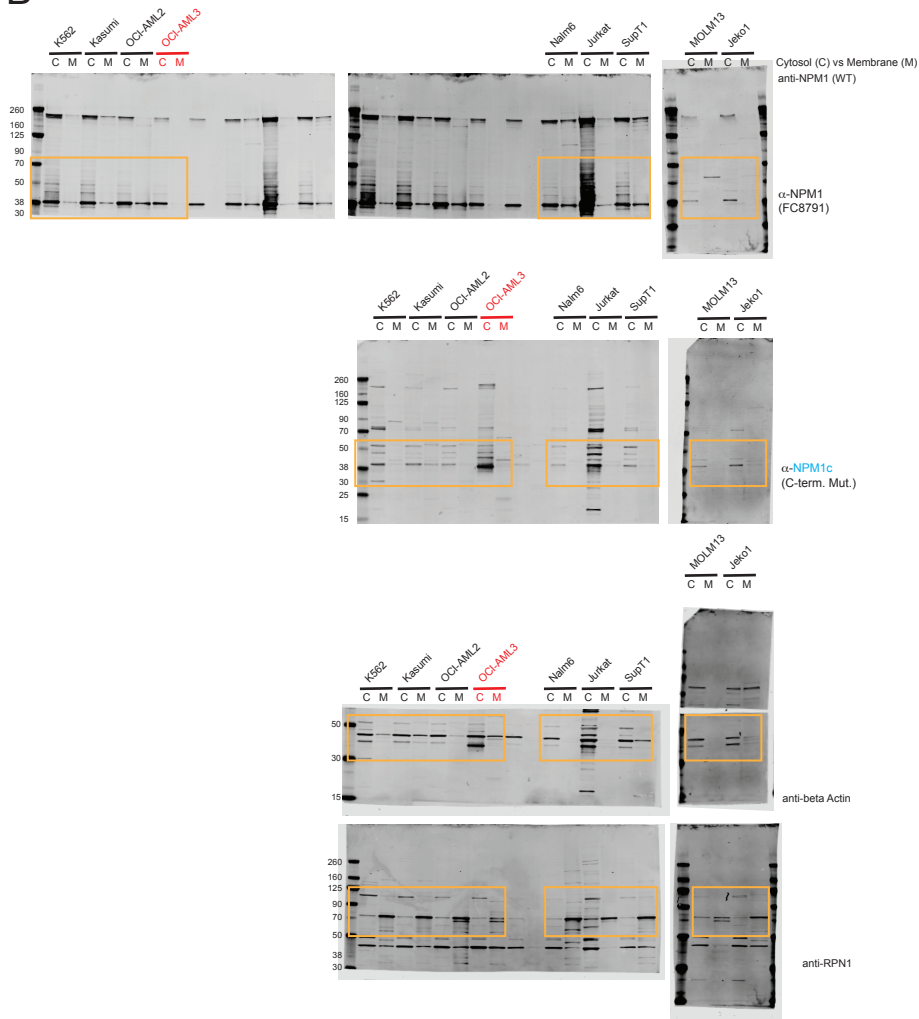

C

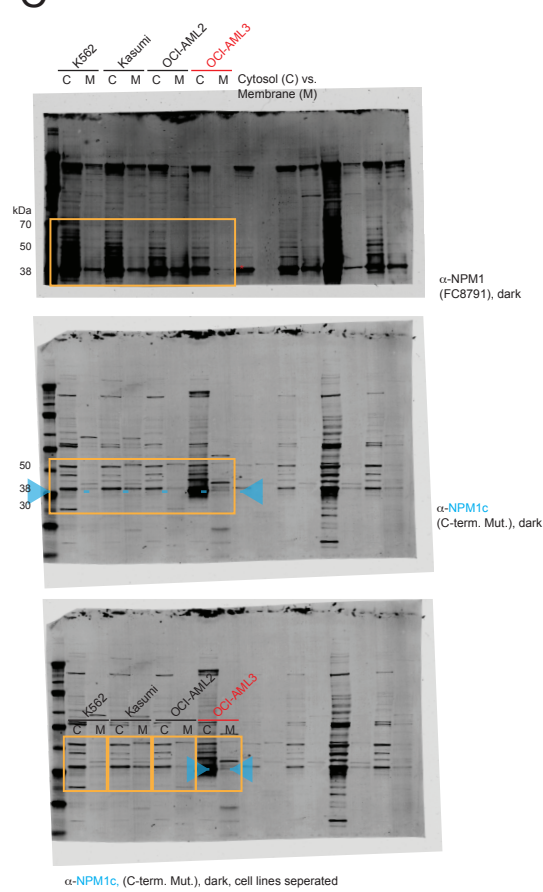

G

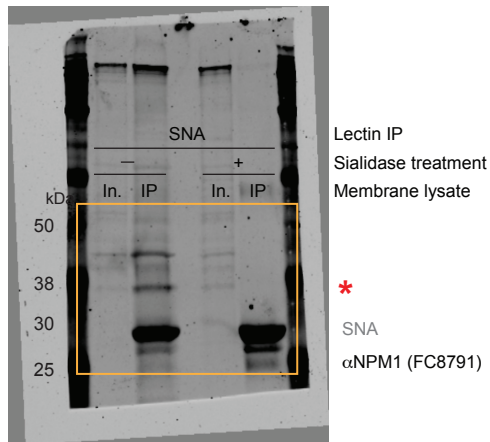

Supplement: Supplementary file 10 — Unprocessed western blots and gels. [file 41587_2025_2648_MOESM10_ESM.pdf]

E

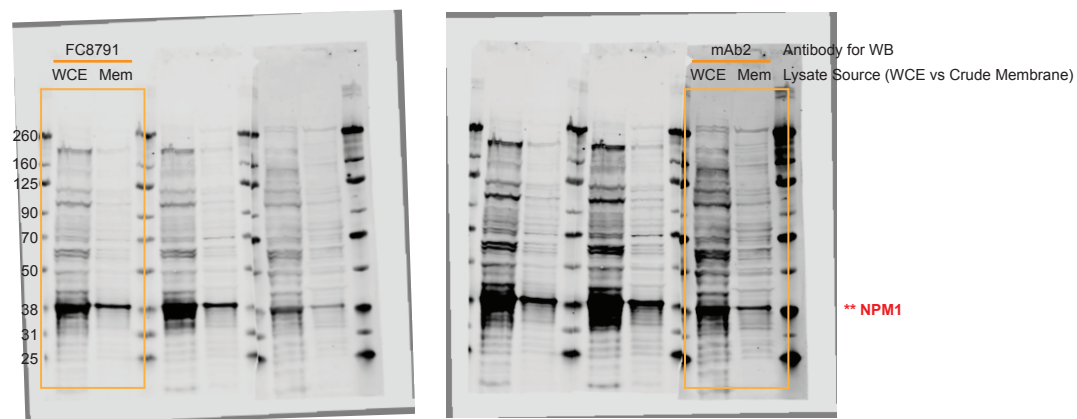

K

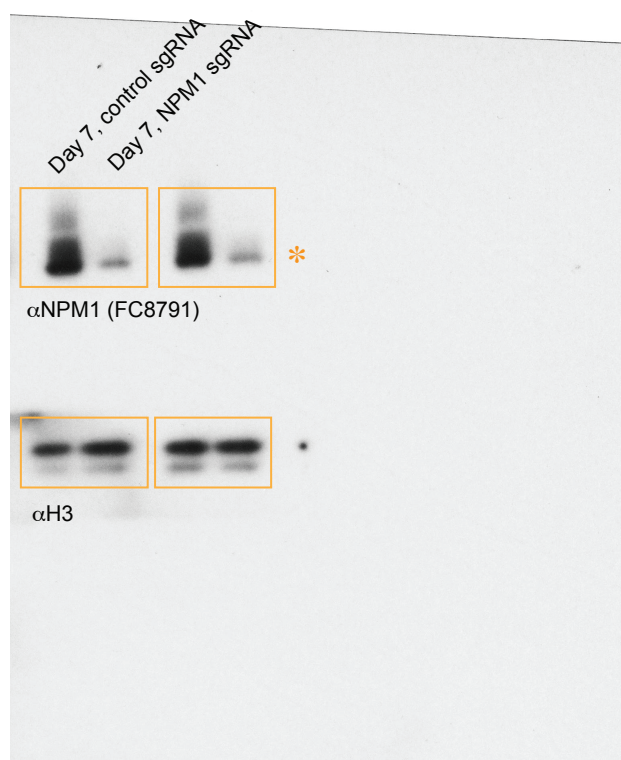

Supplement: Supplementary file 11 — Unprocessed western blots and gels. [file 41587_2025_2648_MOESM11_ESM.pdf]
