# Supplementary material for: Treatment of acute myeloid leukemia models by targeting a cell-surface RNA-binding protein
Source: Nat Biotechnol. Author manuscript; Available in PMC 2025 Dec 24. (PMC7618518; doi:10.1038/s41587-025-02648-2)
Supplement: Supplementary file 1 [file EMS211821-supplement-Supplementary_Material.pdf]

Tables

Table S1. Statistics collected from super-resolution reconstructions.

Table S2. Cell-surface proximity labeling proteomics.

Table S3. DNA primer names and sequences.

Table S4. The 47 human and mouse solid cancer models profiled.

Figure Legend

Figure 1. Full-length NPM1 forms nanoclusters on the surface of cells with other RBPs.

A. Confocal microscopy images of OCI-AML3 cells presented as one z-slice (all images are z-slices unless otherwise specified). Cells in the top row were stained with the anti-NPM1 (FC8791) antibody after fixation and permeabilization while the bottom row of cells were stained while alive. 4′,6-diamidino-2-phenylindole (DAPI) in shown in blue. Scale bars are 10 µm. Nucleoli are indicted with white lines. Representative of three individual replicates.
B. Confocal microscopy images of OCI-AML3 stained alive with anti-NPM1 (FC8791) and biotinylated WGA. DAPI shown in blue. Scale bars are 10 µm. Representative of three individual replicates.
C. Western blot of K562 cells first labeled with sNHS-SS-biotin. NPM1 immunoprecipitation (IP, left) or streptavidin IP (right) were performed and stained with anti-NPM1 or streptavidin. Molecular weights are shown in kilodaltons (kDa). Red asterisks mark the expected weight of full-length NPM1. Representative of three individual replicates.
D. HL-60 cells stained live with anti-NPM1 antibody and subsequently fixed. Shown are diffraction-limited (DL) widefield microscopy images (left) or super-resolution reconstructions (SR, right), presented as 2D histograms (bin size: 17.7 nm). Scale bar 10 µm. For SR images, the calibration bar indicates the number of localizations per histogram bin.
E. A zoomed view of the indicated region in (D). Scale bar 1.42 µm.
F. OCI-AML3 cells stained and analyzed as in (D).
G. A zoomed view of the indicated region in (F). Scale bar 1.42 µm.
